# Control regions for chromosome replication are conserved with respect to sequence and location among *Escherichia coli* strains

**DOI:** 10.3389/fmicb.2015.01011

**Published:** 2015-09-24

**Authors:** Jakob Frimodt-Møller, Godefroid Charbon, Karen A. Krogfelt, Anders Løbner-Olesen

**Affiliations:** ^1^Department of Biology, Section for Functional Genomics and Center for Bacterial Stress Response and Persistence, University of CopenhagenCopenhagen, Denmark; ^2^Department of Microbiology and Infection Control, Statens Serum InstitutCopenhagen, Denmark

**Keywords:** *E. coli*, *oriC*, non-coding replication control regions, *datA*, *DARS1* and *DARS2*, fitness *in vitro* and *in vivo*

## Abstract

In *Escherichia coli*, chromosome replication is initiated from *oriC* by the DnaA initiator protein associated with ATP. Three non-coding regions contribute to the activity of DnaA. The *datA* locus is instrumental in conversion of DnaA^ATP^ to DnaA^ADP^ (*datA* dependent DnaA^ATP^ hydrolysis) whereas DnaA rejuvenation sequences 1 and 2 (*DARS1* and *DARS2*) reactivate DnaA^ADP^ to DnaA^ATP^. The structural organization of *oriC, datA, DARS1*, and *DARS2* were found conserved among 59 fully sequenced *E. coli* genomes, with differences primarily in the non-functional spacer regions between key protein binding sites. The relative distances from *oriC* to *datA, DARS1*, and *DARS2*, respectively, was also conserved despite of large variations in genome size, suggesting that the gene dosage of either region is important for bacterial growth. Yet all three regions could be deleted alone or in combination without loss of viability. Competition experiments during balanced growth in rich medium and during mouse colonization indicated roles of *datA, DARS1*, and *DARS2* for bacterial fitness although the relative contribution of each region differed between growth conditions. We suggest that this fitness advantage has contributed to conservation of both sequence and chromosomal location for *datA, DARS1*, and *DARS2*.

## Introduction

**Figure 1:**
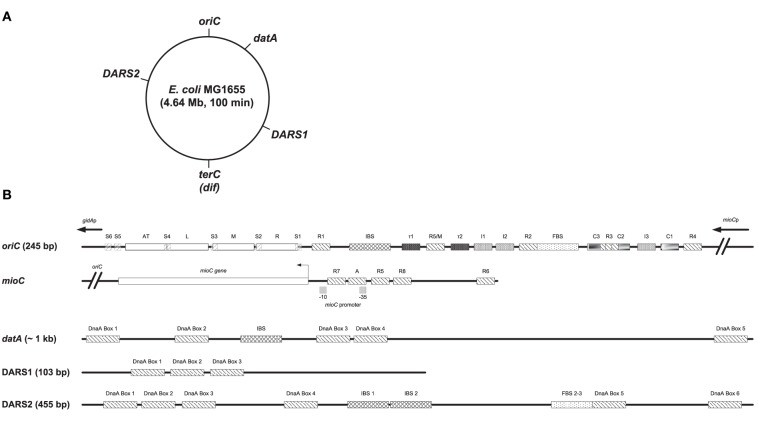
**Structures of ***oriC, mioC*** promoter, ***datA, DARS1***, and ***DARS2***. (A)** The MG1655 chromosome is shown as a circle and the locations of *oriC* (at the genome map position of 84.6 min), *datA* (at 94.6 min), *DARS1* (at 17.5 min), *DARS2* (at 64.0 min), and *terC*/*dif* (around 36 min) are indicated. **(B)** Basic structures of *oriC*, the *mioC* promoter, *datA, DARS1*, and *DARS2* are schematically shown. Both high- to medium affinity sites (R1, R4, and R2) that binds both DnaA^ATP^ and DnaA^ADP^, lower affinity sites (R3, R5/M, I1, I2, I3, C1, C2, C3, τ1, and τ2), and Single-stranded DnaA-ATP box 1–6 (S1-6) that only binds DnaA^ATP^ are indicated in *oriC* (see text for details). The minimal *oriC* sequence (245 bp) is defined to end just to the left of the 13-mer termed L and to the right of DnaA Box R4. Note that the R3 DnaA box overlaps with DnaA box C3 and C2 in *oriC*. DnaA boxes in the *mioC* promoter are indicated as described by Hansen et al. (2007), with DnaA Box R5 and R6 being high affinity sites, while DnaA Box R7, R8, and A are lower affinity sites. *DARS1* contains 3 DnaA boxes, *DARS2* contains 6 DnaA boxes an IBS and a FBS, and *datA* contains 5 DnaA boxes and an IBS. IBS, IHF-binding site; FBS, FIS-binding site. Figure is not to scale.

In *Escherichia coli* chromosome replication is initiated from a single origin, *oriC*, and proceeds bi-directionally until the two replication forks meet at terminus of replication (*terC*). The initiator protein DnaA belongs to the AAA^+^ (ATPases Associated with diverse Activities) proteins. DnaA can bind ATP and ADP with similar high affinities (Skarstad and Katayama, 2013), but only DnaA bound to ATP is able to initiate DNA replication (Sekimizu et al., 1987). Different recognition sites for DnaA has been identified in *oriC*; three high to medium affinity sites (R1, R4, and R2) that binds both DnaA^ATP^ and DnaA^ADP^ (Fuller et al., 1984), and multiple lower affinity sites (R3, R5/M, I1, I2, I3, C1, C2, C3, τ1, and τ2) (McGarry et al., 2004; Kawakami et al., 2005; Rozgaja et al., 2011) (Figure 1). Only DnaA^ATP^ is capable to bind to low-affinity sites (McGarry et al., 2004; Kawakami et al., 2005; Rozgaja et al., 2011), and single stranded DnaA boxes (Speck and Messer, 2001). Binding of the Fis protein to *oriC* is reported to both inhibit initiation of replication (Wold et al., 1996; Ryan et al., 2004; Riber et al., 2009), stimulate initiation (Flåtten and Skarstad, 2013), or have no effect on initiation (Margulies and Kaguni, 1998), while the binding of integration host factor (IHF) plays a central role in forming an optimal complex (Ryan et al., 2002; Keyamura et al., 2007; Ozaki and Katayama, 2012). Binding of DnaA^ATP^ to both high- and low affinity DnaA boxes in *oriC* are proposed to result in a oligomeric DnaA structure, which assisted by IHF leads to duplex opening in the AT-rich region, i.e., open complex formation (Skarstad and Katayama, 2013). Following duplex opening the helicase DnaB is loaded onto the now single-stranded DNA by the help of DnaA, which leads to further duplex opening and assembly of the replisome (Skarstad and Katayama, 2013).

Initiation of replication is a highly regulated process in *E. coli*. Replication begins essentially simultaneously at all cellular origins (Skarstad et al., 1986), i.e., in synchrony and only once per cell cycle. The tight control is primarily ensured by the oscillation of DnaA^ATP^ that has a temporal increase around the time of initiation, and decreases rapidly thereafter (Kurokawa et al., 1999). Following initiation, *oriC* is temporarily inactivated by the binding of SeqA to hemi-methylated GATC-sites (Campbell and Kleckner, 1990; Lu et al., 1994). This sequestration lasts for about 1/3 of the doubling time and provides a time period for RIDA (Regulatory Inactivation of DnaA) and DDAH (*datA*-dependent DnaA^ATP^ hydrolysis) to hydrolyse DnaA^ATP^ to DnaA^ADP^. In RIDA, the Hda protein, in association with the DNA-loaded β-clamp (DnaN), activates the intrinsic ATPase activity of DnaA, which converts DnaA^ATP^ into DnaA^ADP^ (Kurokawa et al., 1999; Kato and Katayama, 2001). DDAH is an IHF dependent hydrolysis of DnaA^ATP^ to DnaA^ADP^, which takes place at the *datA* locus (Kasho and Katayama, 2013). *datA* contain five DnaA boxes as well as an IHF-binding site (Nozaki et al., 2009; Kasho and Katayama, 2013) (Figure 1). Common for both RIDA and DDAH is that both processes lower the DnaA^ATP^/DnaA^ADP^ ratio to counter unwanted re-initiation of replication. At later stages in the cell cycle the DnaA^ATP^ level must increase past a critical level for a new round of initiation of replication. This is done by rejuvenation of DnaA^ADP^ to DnaA^ATP^ at the *DARS1* and *DARS2* loci, where rejuvenation at the *DARS2* locus is dependent on IHF and Fis (Kasho et al., 2014). In addition *de novo* synthesis of DnaA, which by and large will be ATP bound because ATP is more abundant than ADP within the cell, will also contribute to the increase in DnaA^ATP^ (Kurokawa et al., 1999). *DARS1* and *DARS2* contain a core of three DnaA boxes (Figure 1). In addition, *DARS1* needs a specific DNA region flanking the core for stimulation of ADP dissociation from DnaA (Fujimitsu et al., 2009), while *DARS2* contains three additional DnaA boxes and requires both Fis binding sites (FBS) 2 and 3, and IHF binding to IHF binding sites (IBS) 1 and 2 be active (Kasho et al., 2014) (Figure 1).

**Figure 2:**
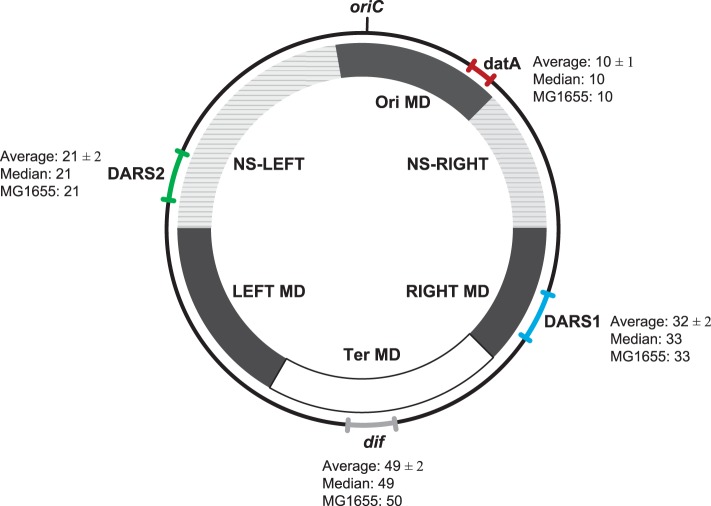
**Location of ***datA, DARS1, DARS2***, and ***dif regions on the E. coli*** genomes**. The range (the relative distance ± the standard deviation in centisomes) in the chromosomal position of *datA* (red), *DARS1* (blue), *DARS2* (green), and *dif* (gray) are shown in the outer circle. For each region the average relative distance with the standard deviation (see Materials and Methods), the median, as well as the chromosomal position in MG1655 (MG1655) are indicated. All distances are given in centisomes. The inner circle schematically shows the location of the different MD- and NS-regions as indicated by Esnault et al. (2007).

Termination of replication occurs in *terC*, a poorly-defined region approximately 180° away from *oriC* (Hill et al., 1987). If an uneven number of homologous recombination events between daughter chromosomes have taken place during replication, the end result will be a chromosome dimer (Sherratt et al., 2004). Resolution takes place at a 28 bp site *dif*, located in *terC* (Sherratt et al., 2004) in a process involving two tyrosine recombinases, XerC and XerD. The XerCD recombinase is activated and delivered at *dif* by the FtsK translocase (Bigot et al., 2005). Numerous forces seem to shape the organization of bacterial chromosomes, and the pattern of these forces on the chromosome is evident at different levels. In both the Gram-negative bacteria *E. coli* (Bergthorsson and Ochman, 1998) and *Salmonella enterica* (Liu and Sanderson, 1995a,b), as well as the Gram-positive bacterium *Lactococcus lactis* (Campo et al., 2004), selective pressure maintains a global architecture of the chromosome, which preserves two replication arms of nearly equal length. In addition to chromosome symmetry further chromosomal constrains are observed in *E. coli*. Four insulated macrodomains (MD) and two less constrained regions called non-structured (NS) regions has been uncovered (Niki et al., 2000; Valens et al., 2004). MDs are defined as regions where DNA interactions occur preferentially, while DNA interactions between the different MDs are highly restricted. NS regions can however interact with both its flaking MDs (Valens et al., 2004). *oriC* and *datA* are contained within the Ori MD, while the Ter MD contains *dif*. The Ori MD is flanked by NS^Right^ and NS^Left^ (where *DARS2* is found) whereas the Ter MD is flanked by the Left MD and the Right MD which contain *DARS1* (Valens et al., 2004) (Figure 2). Several observations indicates that the MDs and NS plays a part in the segregation of sister chromatids and the mobility of chromosomal DNA. The Ori MD is centered on a centromere-like 25 bp sequence designated *migS*, which affects *oriC* positioning during chromosome segregation (Yamaichi and Niki, 2004; Fekete and Chattoraj, 2005). Furthermore, movement of the Ter MD is maintained by several factors including the MatP/*matS* system (Mercier et al., 2008), and ZapA, ZapB, and FtsZ (Espeli et al., 2012; Buss et al., 2015).

Despite of the restraints on the *E. coli* K-12 chromosome, the size of genomes of other *E. coli* species varies from 4.6 to 5.7 Mb, indicating that horizontal gene transfer and genome reductions frequently takes place (Leimbach et al., 2013). A very dynamic genome structure underlies the metabolic and phenotypic diversity of *E. coli*. The genome of a bacterial species can be grouped into two categories. The core genome contains genes present in all strains, while the flexible genome comprises genes that are present in only a few strains or unique to single isolates (Medini et al., 2005). The pan-genome of a bacterial species is the combination of the core genome and the flexible genome (Medini et al., 2005). A typical *E. coli* genome has approximately 5000 genes, where roughly 2200 genes represent the core genome (Rasko et al., 2008). *E. coli* has a very large pan-genome (>18,000 genes), which grows for each new genome sequenced (Medini et al., 2005; Rasko et al., 2008). This indicates that there is a great diversity in gene content between *E. coli* species. Nevertheless, comparison of bacterial chromosomes from related genera revealed a conservation of organization (Eisen et al., 2000). For instance, even though *E. coli* and *Salmonella typhimurium* diverged from a common ancestor about 140 million years ago their genetic maps are extensively superimposable (Groisman and Ochman, 1997).

Here we report a conserved chromosomal position of the non-coding regions *datA, DARS1*, and *DARS2* relative to *oriC* in *E. coli*. In addition, we report that the structural organization of *oriC, datA, DARS1*, and *DARS2* regions are conserved in all *E. coli* strains analyzed. Furthermore, we demonstrate that even though the loss of *datA, DARS1*, or *DARS2* did not result in a measurable reduction in growth rate, the mutant cells had a lower fitness than wild-type when tested under laboratory conditions or in mice.

## Materials and methods

### Growth conditions

Cells were grown in Luria–Bertani Broth (LB) or AB minimal medium supplemented with 0.2% glucose, 10 μg/ml thiamine, and when indicated 0.5% casamino acids. MacConkey agar was used for mice experiments. All cells were cultured at 37°C. When necessary, antibiotics were added to the following concentrations: kanamycin, 50 μg/ml; streptomycin, 100 μg/ml; chloramphenicol, 20 μg/ml; ampicillin, 150 μg/ml.

### Bacterial strains and plasmids

A spontaneous streptomycin resistance mutant of MG1655 (ALO1825) was obtained by plating an overnight culture on streptomycin-plates, resulting in MG1655 StrR (ALO4292) (see Table S1 for used strains).

The *DARS2* region containing DnaA box I-III was replaced with the cat gene in MG1655 by the lambda red procedure (Datsenko and Wanner, 2000), resulting in the Δ*DARS2*::*cat* mutant (ALO4254). Briefly, DNA fragments were PCR amplified using modified primers MutH-9 (5′-TCACAGTTATGTGCAGAGTTATAAACAGAGGAAGGGGTG GATAGCCGTTTCGATTTATTCAACAAAGCCACG-3′) and MutH-10 (5′-CTACGGAATTACTACGGGAAAACCCGGAGC ATTCTGAATAAGCCCGATATGCCAGTGTTACAACCAATTA ACC-3′), where the underlined sequence will anneal to pKD3 (Datsenko and Wanner, 2000). Each deletion was verified by PCR. The *DARS2* deletion was moved from ALO4254 to ALO4292 by P1 transduction using established procedures (Miller, 1972) and by selection for chloramphenicol resistance, resulting in ALO4310. The *cat* gene was removed from ALO4310 by pCP20, according to a method described previously (Cherepanov and Wackernagel, 1995), resulting in ALO4312.

The *DARS1* region was replaced with the *cat* gene in ALO4292 harboring pKD46, as described above, resulting in the Δ*DARS1*::cat mutant (ALO4313). DNA fragments were PCR amplified using pKD3 as template and primers *DARS1*_pKD3_FW (5′-TACATAAACCTTGCCT TGTTGTAGCCATTCTGTATTCGATTTATTCAACAAAGCCA CG-3′) and *DARS1*_pKD3_RV (5′-AAAACAGTTCATCAC CATAATATTTCTGATACAGCGTAAAGCCAGTGTTACAACC AATTAACC-3′) using pKD3 as a template. Each deletion was verified by PCR. The double deletion of *DARS1* and *DARS2* was obtained in the same background by moving the *DARS1* deletion from ALO4313 into the *cat* sensitive ALO4312 by P1 transduction, resulting in ALO4315.

The Δ*datA::kan* allele was obtained from RSD428 (Kitagawa et al., 1998). The *datA* deletion was moved into ALO4292 and ALO4315 by P1 transduction, selecting for kanamycin, resulting in ALO4331 and ALO4511 respectively. Each deletion was verified by PCR.

*lacZ*::Tn*5*::*kan* was moved from MC1000 F' lacI^q^, *lacZ*::Tn*5* (laboratory stock) to MG1655 by P1 transduction and selecting for kanamycin, resulting in ALO1257. *DARS1* and *DARS2* were deleted in ALO1257 (as described above) to give ALO4618 and ALO4619. The *cat* gene was removed from ALO4618 and ALO4619 by pCP20, before transformation of pALO75 (Løbner-Olesen et al., 1987) for investigation of β-galactosidase synthesis from the *mioC* promoter. Strain RB210 (MC1000 carries a *dnaA-lacZ* translational fusion on phage λRB1 integrated at *att*λ (Braun et al., 1985). λRB1 was transduced from RB210 to ALO1257, resulting in ALO1265. The deletion of *DARS2* or *datA* was done as described above and resulted in strains ALO4626 and ALO4627, respectively, for investigation of β-galactosidase synthesis from the *dnaA* promoter.

### Flow cytometry

Flow cytometry was performed as described previously (Løbner-Olesen et al., 1989) using an Apogee A10 instrument. For each sample, a minimum of 30,000 cells were analyzed. Numbers of origins per cell and relative cell mass were determined as described previously (Løbner-Olesen et al., 1989).

The distribution of origins per cell was measured after treating exponentially growing cells with rifampicin and cephalexin for 4 h. Rifampicin block initiation of replication, while cephalexin will block cell division. The average number of chromosomes per cell will therefore be equivalent to the number of *oriC's* present in the cell at the time the drugs were added.

### Relative distance

The relative distance between *oriC* and *DARS1, DARS2, datA*, and *dif* were calculated in centisomes (see equation below). Each *E. coli* chromosome is by definition 100 centisomes. The relative distance is set as the distance in base pairs between *oriC* and the region of interest divided by the size of the genome in base pairs of the investigated *E. coli* strain. There are two distances to *dif*, one for each replication arm. In this study only the shortest replication arm is presented, while the distance of the longest replication arm by default is the sum of the shortest replication arm substracted from 100.

$$\begin{array}{l} Relative\;distance\;in\;centisomes=\\ \left(\frac{\left[Position\;of\;oriC\right]-[Position\;of\;region\;of\;interest]}{\left[Chromosome\;size\right]}\right)*100 \end{array}$$

To calculate the relative distance the chromosome needs to be fully assembled. For this the sequence of 70 fully assembled *E. coli* chromosomes from The European Nucleotide Archive (http://www.ebi.ac.uk/genomes/bacteria.html) were obtained and analyzed. Two times two strains were uploaded under the same strain name, i.e., W and ST540. We denote them Wa (uploaded under sequence CP002185), Wb (uploaded under sequence CP002967), ST540a (uploaded under sequence CP002185), and ST540b (uploaded under sequence CP002967). Wa are identical to Wb, why Wa was used, while ST540b was used as ST540a was excluded (see below). BL21-DE3 and BL21-Gold were excluded for being deviates of B str. REL606, while KO11, KO11FL, and LY180 were excluded for being deviates of W.

Six *E. coli* genomes were found to have relative distances, which were two times the standard deviation or more away from the average (Table S2). Of these W3110, MC4100, and strain ST540a were excluded. ST540a and MC4100 has 20% or more imbalance between the length of the two replication arms, which have been shown to give abnormal cells that was dependent on the RecBC-dependent homologous recombination for viability (Esnault et al., 2007), why they were excluded. W3110 is disqualified due to a known inversion around *oriC* (Hayashi et al., 2006), which explains the altered relative distances compared to the *E. coli* average.

The final dataset comprised of 59 fully assembled *E. coli* genomes (Table S3). The position and sequence of *oriC, DARS1, DARS2, datA*, and *dif* are known in MG1655, but not annotated in the dataset. We therefore choose to annotate the regions in 58 remaining *E. coli* genomes. The sequence of each of the regions (see Supplementary Data) from MG1655 where therefore aligned with the chosen *E. coli* genomes to obtain the chromosomal position and sequence of the region in each individual *E. coli* genomes.

### Mutation frequency

The mutation frequency was estimated for intergenic regions. Regions between protein-coding genes of more than 300 bp were selected in MG1655. These regions were trimmed for 100 bps on each side, to avoid conserved promoter-regions, and blasted against the 58 remaining *E. coli* genomes. If not present in the entire dataset the intergenic region was discharged, resulting in 109 regions. Of these 13 intergenic regions contained known conserved sRNA or tRNA's, why they were removed, resulting in 96 intergenic regions. Each intergenic region was aligned and number of nucleotides that were not present in every genome was calculated to give a mutation frequency.

### Competition experiment in LB

The fitness of Δ*DARS1*, Δ*DARS2*, Δ*DARS1* Δ*DARS2*, and Δ*datA* compared to the wild-type were investigated during direct competition in LB medium. The competing strains were inoculated pairwise at an approximate concentration of (10^7^ CFU/mL) each. The populations were propagated by continuously transfers in LB medium. Samples from each population were taken at 10-generation intervals. Each sample was diluted in 0.9% NaCl and plated on LB plates with appropriate antibiotics. To distinguish the various *E. coli* strains, dilutions were plated on LB plates containing no antibiotic, kanamycin, or chloramphenicol. All plates were incubated for 18–24 h at 37°C prior to counting. When necessary to distinguish strains, 100 colonies from plates containing no antibiotic were toothpicked onto LB plates containing kanamycin or LB plates containing chloramphenicol.

### Mouse colonization experiments

The specifics of the streptomycin-treated mouse model used to compare the large intestine colonizing abilities of *E. coli* strains in mice have been described previously (Leatham et al., 2005; Leatham-Jensen et al., 2012). Briefly, Six-to-eight-week-old, outbreed female CD-1 (Charles River Laboratories, Netherlands) mice were given drinking water containing streptomycin sulfate (5 g/l) for 24 h to eliminate resident facultative anaerobic bacteria (Miller and Bohnhoff, 1963). Mice were orally fed 100 μL of 20% (wt/vol) sucrose containing 10^6^ CFU LB grown *E. coli* strains. The number of *E. coli* colonizing the mouse large intestine is reflected in the mouse feces, which is why fecal counts are used to estimate the various *E. coli* strains' ability to colonize the mouse intestine (Leatham-Jensen et al., 2012). After ingesting the bacterial suspension; feces was collected after 24 h, and as indicated. The mice were caged in groups of three mice, and cages were changed weekly. Mice were marked so they could be isolated and fecal pellets could be collected from each individual mouse. Mice were given fresh drinking water containing streptomycin sulfate (5 g/l) each day. Each fecal sample was homogenized in 1% Bacto tryptone (Difco Laboratories, NJ, USA), diluted in the same medium, and plated on MacConkey agar plates with appropriate antibiotics. When appropriate, 1 ml of a fecal homogenate (sampled after the feces had settled) was centrifuged at 12,000 X *g*, resuspended in 100 μL of 1% Bacto tryptone, and plated on a MacConkey agar plate with the appropriate antibiotics. This procedure increases the sensitivity of the assay from 10^2^ CFU/gram of feces to 10 CFU/per g of feces. To distinguish the various *E. coli* strains in feces, dilutions were plated on lactose MacConkey agar containing either streptomycin, streptomycin and kanamycin, or streptomycin and chloramphenicol. All plates were incubated for 18–24 h at 37°C prior to counting. When necessary to distinguish strains, 100 colonies from plates containing streptomycin were toothpicked onto MacConkey agar plates containing streptomycin and kanamycin or onto MacConkey agar plates containing streptomycin and chloramphenicol. Ethics approval statement; 2007/561-1430.

### β-Galactosidase assays

Cells were grown exponentially at 37°C in AB minimal medium supplemented with casamino acids, and β-galactosidase activities were measured as described by Miller (1972).

## Results

### Conserved relative distance from *oriC* to *datA, DARS1, DARS2*, and *dif* in *E. coli*

Bergthorsson and Ochman (1998) suggested that there is an evolutionary pressure on keeping the *E. coli* chromosome symmetric, so an approximately equal length of the two replication arms are maintained. The non-coding regions *DARS1, DARS2*, and *datA* are all indirectly involved in initiation of replication at *oriC* as they modulate the activity and for *datA* also the amount of DnaA available for initiation in a dosage dependent manner. The replication-associated gene dosage of each region relative to *oriC* changes with growth rate and is given by the formula N_x_/N_*oriC*_ = 2^([*C* × (1−*x*) +*D*]∕τ)^ where x is the relative distance from *oriC*, C is the replication period, D is the time following termination of replication until cell division, and τ is the doubling time (Bremer and Churchward, 1977). We therefore decided to investigate if there was any evolutionary pressure on their chromosomal position relative to *oriC*. The genome size of *E. coli* varies from 4.6 to 5.7 Mb (Leimbach et al., 2013). Thus, to compare chromosomal positions between genomes with up to 1 Mb difference we calculated a relative distance from *oriC* (see Materials and Methods) while using MG1655 as reference strain. The replication terminus is not as well defined as the origin of replication, this is why *dif* was chosen to represent *terC* (Hendrickson and Lawrence, 2007).

Although the study was limited to 59 “closed” *E. coli* genomes, the dataset includes a wide variety of different *E. coli* (see Table S3). Pathogenic *E. coli* strains are categorized into pathotypes (Kaper et al., 2004). The dataset includes four pathotypes, which are associated with diarrhea, namely shiga toxin-producing *E. coli* (STEC)/enterohemorrhagic *E. coli* (EHEC), enterotoxigenic *E. coli* (ETEC), enteropathogenic *E. coli* (EPEC), and enteroaggregative *E. coli* (EAEC). In addition to the intestinal pathogens two *E. coli* associated with the inflammatory bowel disease Crohn's disease were also included. In contrast to intestinal pathogenic *E. coli* (IPEC), which are obligate pathogens, extraintestinal pathogenic *E. coli* (ExPEC) are facultative pathogens which belong to the normal gut flora of a certain fraction of the healthy population where they live as commensals (Köhler and Dobrindt, 2011). The dataset contains ExPEC associated with neonatal-meningitis, asymptomatic bacteriuria, acute cystitis, the multidrug resistant ST131, as well as several uropathogenic *E. coli* (UPEC). In addition to human pathogenic *E. coli* strains, several *E. coli* strains isolated from the feces of healthy individuals (human commensals) are included. Apart from numerous common *E. coli* laboratory strains the dataset is concluded by three *E. coli* strains shown to be pathogenic in animals (avian pathogenic *E. coli* (APEC), and porcine enterotoxigenic *E. coli*) as well as an *E. coli* isolated from a toxic-metal contaminated site (for references see Table S3).

The chosen *E. coli* genome dataset had a median genome size of 5,095,204 bp, spanning from 3,976,195 bp (MDS42) to 5,697,240 bp (O26:H11 str. 11368). MDS42 is a “man-made” reduced *E. coli* K-12 genome derived from MG1655, which was constructed to identify non-essential genes (Pósfai et al., 2006). The smallest non-lab constructed *E. coli* chromosome was BW2952 with 4,578,159 bp. Due to the great diversity in both origin of isolation and genome size we believe that the dataset will be representative of *E. coli* as a whole.

Despite the large differences in genome size between *E. coli* strains, we found approximately the same relative distance from *oriC* to *dif, datA, DARS1*, and *DARS2*, respectively (Figure 2). This observation points to a conserved chromosomal organization. This organization is further conserved at the replichore level as, *DARS2* was always found on one replichore, while *datA* and *DARS1* were always found on the other replichore. *dif* was found at the chromosomal position opposite of *oriC*, which indicates that both replications arms were of approximately equal length in accordance with data from Bergthorsson and Ochman (1998).

### *E. coli* chromosome symmetry

**Figure 3:**
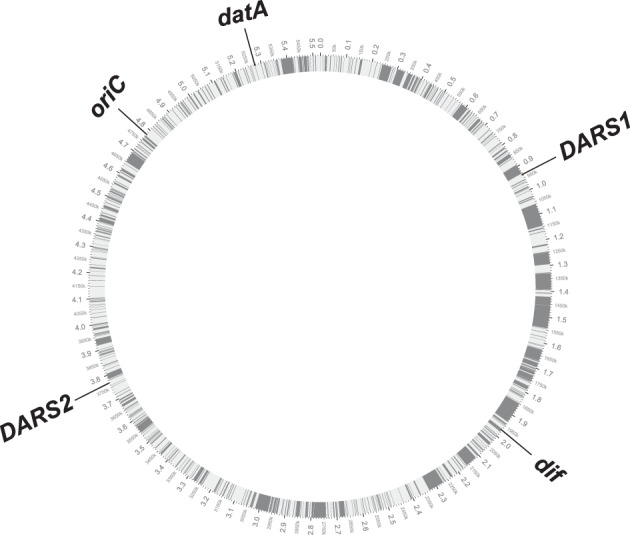
**Circular genome map of O157:H7 EDL933 compared with MG1655**. The outer circle shows the genomic backbone of the O157:H7 EDL933 genome with the chromosomal position of *oriC, datA, DARS1, DARS2*, and *dif* annotated. The inner circle displays in white the common 4.1 Mb backbone between the two *E. coli* genomes, while the gray bars illustrates the distribution of the 1.34 Mb DNA unique to O157:H7.

The conserved position of *DARS1, DARS2, datA*, and *dif* relative to *oriC* in the tested 59 *E. coli* genomes, suggests that new DNA obtained by horizontal gene transfer has been equally distributed between the two replication arms, but also between the different regions on each of the replication arms. Strain O157:H7 EDL933 that has a genome size of 5.53 Mb, i.e., about 0.9 Mb bigger than the laboratory strain MG1655, exemplifies this (Figure 3). MG1655 and O157:H7 EDL933 shares a common 4.1 Mb backbone, which is co-linear except for one 422-kilobase inversion spanning the replication terminus (Perna et al., 2001). The differences between the two genomes are reflected in K-islands (0.53 Mb), which is the DNA present only in MG1655 and O-islands (1.34 Mb), which is unique to O157:H7 EDL933 (Perna et al., 2001). When a circular genome map of O157:H7 EDL933 is compared to MG1655 the 1.34 Mb DNA unique to O157:H7 EDL933 is not only distributed between the two replication arms but as expected also among the *cis*-acting regions for regulation of initiation of replication (gray boxes; see Figure 3).

The *E. coli* strain MDS42 (Pósfai et al., 2006) contains a 14.0% reduced genome relative to it's the parental MG1655. However, it maintained a similar relative distance from *oriC* to *DARS1, DARS2, datA*, and *dif* as the parental strain (see Table S3), i.e., the non-essential DNA lost from MG1655 was distributed between the different non-coding *cis*-acting regions.

### Conservation of *oriC, DARS1, DARS2* and *datA* regions

Only a few genomes showed 100% sequence identity of the *oriC, DARS1, DARS2*, and *datA*-regions to those of MG1655. The comparison between the nucleotide sequences from the 59 different *E. coli* genomes is found in the Supplementary Material (Supplementary Figures S12–S15).

In order to estimate the mutation pressure on *oriC, datA, DARS1*, and *DARS2* we calculated the mutation frequency for intergenic regions in *E. coli* (see Materials and Methods). It was found to be 6 mutations per 100 ± 3 bp. Neither of the *oriC, datA, DARS1*, or *DARS2* regions differed significantly from this average frequency (Not shown). However, the vast majority of changes observed were found in spacer regions whereas binding sites for key proteins were conserved among all genomes (Table 1) which underlines their important role for cell cycle control.

**Table 1:** **Sequence deviations in conserved regions of ***oriC, DARS1, DARS2*** and ***datA*****.

**Region**	**Identity**	**Functional site^a^**	**No. of sequences deviating from MG1655^b^**	**See Figures**
*oriC*	Conserved	13-mer L 13-mer M DnaA Box R1 DnaA Box R2 DnaA Box R5 DnaA Box τ1 DnaA Box τ2 DnaA Box I3 DnaA Box C1 DnaA Box C2 SSDA Box 3 SSDA Box 4 SSDA Box 6 AT		
	Not conserved	13-mer R	6/59	S1
		SSDA Box 1	1/59	S2
		SSDA Box 2	5/59	S2
		SSDA Box 5	1/59	S2
		DnaA Box I1	6/59	S3
		DnaA Box I2	1/59	S4
		DnaA Box C3/R3	10/59	S5
		DnaA Box R4	1/59	S6
		IBS	3/59	S7
*DARS1*	Conserved	DnaA Box 1 DnaA Box 2 DnaA Box 3		
*DARS2*	Conserved	DnaA Box 1 DnaA Box 2 DnaA Box 3 DnaA Box 4 DnaA Box 5 DnaA Box 6 IBS-1		
	Not conserved	FBS-2	3/59	S8
		FBS-3	9/59	S9
		IBS-2	4/59	S10
*datA*	Conserved	DnaA Box 1 DnaA Box 2 DnaA Box 3 DnaA Box 4 DnaA Box 5		
	Not conserved	IBS	12/59	S11

a *FBS, Fis Binding Site; IBS, IHF Binding Site; SSDA, Single stranded binding site for DnaA^ATP^*.

b *Number of isolates within the dataset which deviates from the region in MG1655*.

#### oriC

In *oriC* both of the AT-rich 13-mer regions L and M were identical among the 59 strains, whereas the R 13-mer varied in the two outer positions (Supplementary Figure S1). Three of the six 6-mer sites present in the AT-rich region (Supplementary Figures S3, S4, S6) that specifies binding of DnaA^*ATP*^ when in the single stranded configuration (Figure 1) were identical to the same regions in MG1655. However, Supplementary Figures S1, S2, S5 carries single nucleotide changes relative to MG1655 in a subset of strains. In five strains a nucleotide alteration was found in the single stranded DnaA-ATP box 2 that demolished a GATC-site, which is the substrate for Dam methyltransferase (Supplementary Figure S2).

The majority of DnaA binding sites in *oriC* (R1, R2, R5, τ1, τ2, I3, C1, and C2) were completely conserved, whereas R3, C3, I1, I2, and R4 binding sites carried differences to the corresponding MG1655 sequences in some strains (Supplementary Figure S12). Controversy about which of DnaA Box R3 and DnaA Box C3 are functional during unwinding of *oriC* exists (Kaur et al., 2014). The DnaA Box R3 overlaps with both DnaA Box C2 and C3 (Figure 1; Supplementary Figure S12). Nonetheless, all three DnaA boxes are included in the present study.

The consensus sequence for the R-box is TTWTNCACA (W is dA or dT and N is any nucleotide) (Schaper and Messer, 1995). In MG1655 DnaA Box I1 differs from the R-box consensus sequence by three nucleotides, while DnaA Box C3 and I2 differ by four nucleotides (Grimwade et al., 2000; Ryan et al., 2002; Rozgaja et al., 2011). We only identified sequence alterations that resulted in an altered identity to the R-box consensus sequences for DnaA binding sites, I2, R3, and R4. The I2 binding site from strain ED1a was found to fit better to the R-box consensus sequence compared to the DnaA box I2 sequence from MG1655 (Supplementary Figure S4). The MG1655 DnaA Box R3 differs from the R-box consensus sequence by one nucleotide, while the 10 *E. coli* strains deviating from the MG1655 DnaA Box R3 sequence deviate from the R-box consensus sequence by two nucleotides (Supplementary Figure S5). Strain 0127:H6 E2348/69 deviates from the R-box consensus sequence by a nucleotide in DnaA Box R4 (Supplementary Figure S6). The change from a dT to a dC in nucleotide position number 4 diminishes the identity to the R-box consensus sequence.

The IHF binding site consensus sequence is WATCAANNNNTTR [W is dA or dT, R is dA or dG, and N is any nucleotide (Hales et al., 1994)]. Three strains were found to differ with respect to the *oriC* IBS (Supplementary Figure S7). O26:H11 str. 11368 was found to have a diminished identity to the IHF consensus sequence compared to MG1655, while both O145:H28 str. RM12761 and O145:H28 str. RM13516 was found to have a better fit. The Fis binding site in *oriC* was found completely conserved in all strains (Supplementary Figure S12).

The nucleotide distances between the protein binding regions were highly conserved between strains. Only O26:H11 str. 11368 and O111:H- str. 11128 lacked a nucleotide in the spacer region between the AT-rich 13-mer termed R and the DnaA Box R1 in *oriC* compared to MG1655 (Supplementary Figure S12).

Based on this analysis it is hard to deduce a hierarchy of the importance of the different DnaA binding sites in *oriC*. Ten strains had changes in the R3/C3 boxes. Whereas the alterations resulted in a R3 box with poorer resemblance to the R-box consensus, this was not the case for C3. Therefore, it is likely that C3 represents the functional DnaA binding site in the replication origins.

#### *DARS1, DARS2*, and *datA*

All DnaA binding sites in *datA, DARS1*, and *DARS2* were completely conserved between the strains analyzed (Table 1) (Supplementary Figures S13–S15). FBS-2 of *DARS2* differed from that of MG1655 in three strains. However, since Fis has the consensus sequences GNNYANNNNNTRNNC (Y is dC or dT, R is dA or dG, and N is any nucleotide) (Finkel and Johnson, 1992) none of the observed differences resulted in a reduced similarity to the Fis consensus sequence (Supplementary Figure S8). For FBS-3 of *DARS2*, four *E. coli* strains differed from MG1655 and had a reduced identity the Fis consensus sequence (Supplementary Figure S9).

The IHF binding site IBS-1 of *DARS2* was identical in all 59 genomes. The sequence variations of IBS-2 of *DARS2* (Supplementary Figure S10) or the IBS in *datA* (Supplementary Figure S11), relative to MG1655 did not change the identity to the IHF consensus sequences. The *datA* region of strains HS and O103:H2 str. 12009 DNA lacked four nucleotides between DnaA Box 1 and DnaA Box 2, while strain 536 lacked a nucleotide between the IBS and DnaA Box 3.

Altogether, these observations suggest that there is a strong selection pressure on maintaining the sequence and spacing of protein binding sites, and thereby functionality of *oriC, DARS1, DARS2*, and *datA*. The majority of the nucleotide differences observed was located in the non-functional spacer regions between the different protein binding sites. The majority of differences found within protein binding sites, did not reduce the identity to the investigated consensus sequence.

### Importance of *datA, DARS1*, and *DARS2* for cell cycle control

Despite of the conservation of the chromosomal positions of *DARS1, DARS2*, and *datA* relative to *oriC*, neither is essential (Kitagawa et al., 1998; Fujimitsu et al., 2009). Cells with and without *datA* were also previously found to have similar doubling times (Kitagawa et al., 1998). We created cells with deletions of *datA, DARS1*, and *DARS2* individually and in various combinations. These cells were viable no matter which combination of *DARS1, DARS2*, and *datA* we deleted. The cellular doubling time was not affected by individual deletions but increased when combinations of *DARS1, DARS2*, and *datA* were deleted (Table 2). Cells carrying Δ*DARS1* Δ*DARS2* and Δ*DARS1* Δ*DARS2* Δ*datA* were found to have the longest doubling time in minimal medium supplemented with glucose and casamino acids, while Δ*DARS1* Δ*datA* and Δ*DARS2* Δ*datA* cells were found to have the longest doubling time in the same medium without casamino acids (Table 2).

**Table 2:** **Cell cycle parameters of mutant strains**.

**Strain/Genotype^a^**	**ABTG + CAA^b^ (Min)**	**Origin/Cell^c^**	**Cell mass^d^**	**Origins/Mass^e^**
Wild-type	40	5.2	1	1
Δ*datA*	41	7.9	1	1.5
Δ*DARS1*	40	4.7	0.9	0.8
Δ*DARS2*	41	4.4	0.9	0.8
Δ*DARS1* Δ*DARS2*	45	3.1	1.1	0.6
Δ*DARS1* Δ*datA*	41	7.2	1	1.4
Δ*DARS2* Δ*datA*	42	6.1	1.1	1.2
Δ*DARS1* Δ*DARS2* Δ*datA*	49	4	1	0.8
	**ABTG**^b^			
Wild-type	88	1.4	1	1
Δ*datA*	86	1.7	0.8	1.2
Δ*DARS1*	85	1.3	1.2	0.95
Δ*DARS2*	87	1.3	1.1	0.95
Δ*DARS1* Δ*DARS2*	91	1.2	1.5	0.9
Δ*DARS1* Δ*datA*	96	1.4	0.9	1.1
Δ*DARS2* Δ*datA*	103	1.4	0.9	1.1
Δ*DARS1* Δ*DARS2* Δ*datA*	91	1.2	1.1	0.9

a *Wild-type is MG1655*.

b *Doubling time in minimal medium supplemented with glucose and casamino acids or minimal medium supplemented with glucose. Each strain has a standard deviation of ± 2 min for growth in ABTG + CAA and ± 3 min for growth in ABTG*.

c *Determined from flow cytometric analysis*.

d *Determined as average light scatter from flow cytometric analysis. Numbers are normalized to 1 for wild-type*.

e *Average fluorescence/average light scatter. Numbers are normalized to 1 for wild-type*.

We used *lacZ* fusions of *dnaA* and *mioC* promoters to assess the effect of *datA, DARS1*, and *DARS2* loss on the cellular DnaA^ATP^/DnaA^ADP^ ratio. The *dnaA* gene is transcribed from two upstream promoters, termed *dnaA1p* and *dnaA2p*. Four DnaA boxes are located between the two promoters, with only one of them containing the stringent consensus sequence (Hansen et al., 1982, 2007; Armengod et al., 1988). Both *dnaA* promoters are negatively regulated by the DnaA protein (Hansen et al., 2007), with DnaA^ATP^ being most efficient in repressing *dnaA* expression (Speck et al., 1999). DnaA^ATP^ also repress the *mioC* promoter located upstream of *oriC* prior to initiation by binding to five DnaA boxes located within and/or close the promoter. Of the five DnaA boxes only one contains the stringent consensus sequence (Figure 1) (Ogawa and Okazaki, 1994; Bogan and Helmstetter, 1997; Hansen et al., 2007). Loss of *datA* resulted in a slight repression of *dnaA* (Table 3). This is in agreement with an increase in the DnaA^ATP^/DnaA^ADP^ ratio, and the *dnaA* promoter being repressed by DnaA^ATP^ (Kitagawa et al., 1998; Speck et al., 1999; Kasho and Katayama, 2013). Loss of *DARS1* led to an increased expression of the *mioC* gene while loss of *DARS2* led to an increased expression of both the *dnaA* and *mioC* genes (Table 3), since both promoters are subject to negative transcriptional control by DnaA^ATP^ (Speck et al., 1999; Hansen et al., 2007). This agrees with *DARS1* and *DARS2* being instrumental in increasing the cellular DnaA^ATP^ level, and that *DARS2* is more efficient than *DARS1* (Fujimitsu et al., 2009).

**Table 3:** **Expression of the ***dnaA*** and ***mioC*** genes**.

**Strain**	***dnaA* expression (SD)^a^**	***mioC* expression (SD)^b^**
Wild-type	100	100
Δ*DARS1*	ND	108 (± 5)
Δ*DARS2*	116 (± 1)	122 (± 4)
Δ*datA*	95 (± 3)	ND

a *Measured in MG1655 using the dnaA-lacZ translational fusion carried on λRB1 (Braun et al., 1985) in strain MG1655 lacZ::Tn5. Numbers are given relative to wild-type expression of 100% corresponding to 46 Miller units. ND, Not determined; SD, Standard deviation*.

b *Measured in MG1655 lacZ::Tn5 using the mioC-lacZ transcriptional fusion carried on plasmid pALO75 (Løbner-Olesen et al., 1987). Numbers are given relative to wild-type expression of 100% corresponding to 302 Miller units. ND, Not determined; SD, Standard deviation*.

**Figure 4:**
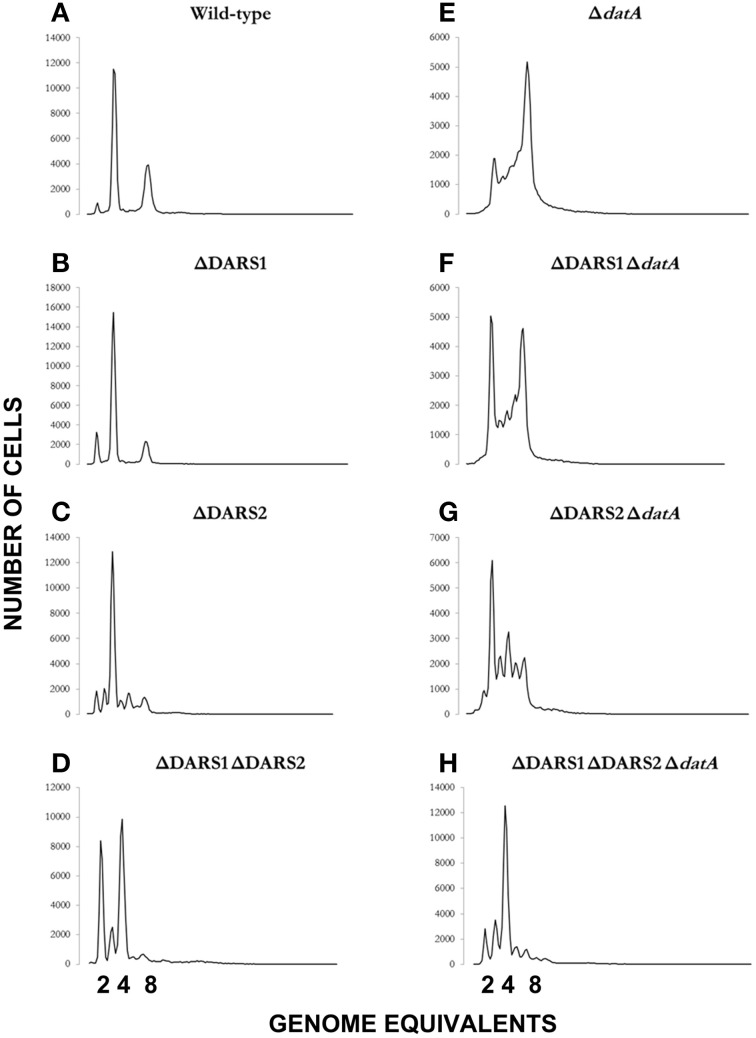
**Cellular origin distribution**. Prior to flow cytometric analysis exponentially growing wild-type and mutant cells was treated with rifampicin and cephalexin. Cells were grown in AB minimal medium supplemented with 0.2% glucose, 10 μg/ml thiamine, and 0.5% casamino acids at 37°C. Wild-type is MG1655 **(A)**; relevant mutations are indicated in individual panels **(B–H)**.

We proceeded to analyze the cell cycle characteristics by flow cytometry (Table 2; Figure 4). Wild-type cells exhibited the expected synchronous initiation pattern with the majority of cells containing 2, 4, or 8 replication origins (Figure 4A) (Skarstad et al., 1986). *datA* deficient cells had an increased origin concentration (origins/mass) (Kitagawa et al., 1998), which resulted both from an increase number of origins per cell and a decreased cell mass (during slow growth only) (Table 2). A high degree of initiation asynchrony was observed for Δ*datA* cells (Figure 4E) (Kitagawa et al., 1998). Cells deficient in *DARS1, DARS2* or both regions had a reduced origin concentration relative to wild-type cells (Table 2; Figures 4B–D) (Fujimitsu et al., 2009). Compared to wild-type, all DARS mutant cells had an increased cell mass during slow growth whereas only the Δ*DARS1* Δ*DARS2* double mutant had increased cell mass during fast growth. Asynchrony of initiation was observed for Δ*DARS2* (Figure 4C) and Δ*DARS1* Δ*DARS2* (Figure 4D) cells, but not for cells carrying the Δ*DARS1* mutation alone (Figure 4B) (Fujimitsu et al., 2009).

Because the *datA* region promotes inactivation of DnaA^ATP^ to DnaA^ADP^, and the DARS regions promote the opposite, i.e., DnaA reactivation, we decided to see whether loss of *DARS1, DARS2* or both could suppress the initiation defect of *datA* cells. Deletion of *DARS1* in Δ*datA* cells only marginally lowered the origin per mass (from 1.5 to 1.4; Table 2). A similar but larger effect was observed when *DARS2* was deleted suggesting that *DARS2* is more efficient than *DARS1* for DnaA rejuvenation. Deleting both *DARS1* and *DARS2* in Δ*datA* cells lowered the origin concentration below wild-type level (Table 2) and also partly restored initiation synchrony (Figure 4H). Overall, these experiments show that loss of rejuvenation activity overcompensates for loss of DDAH. This may be explained by the RIDA process, which being active in the triple mutant so that DnaA^ATP^ to DnaA^ADP^ conversion is still ongoing.

### *DARS1* and *DARS2* are required for mouse colonization

**Figure 5:**
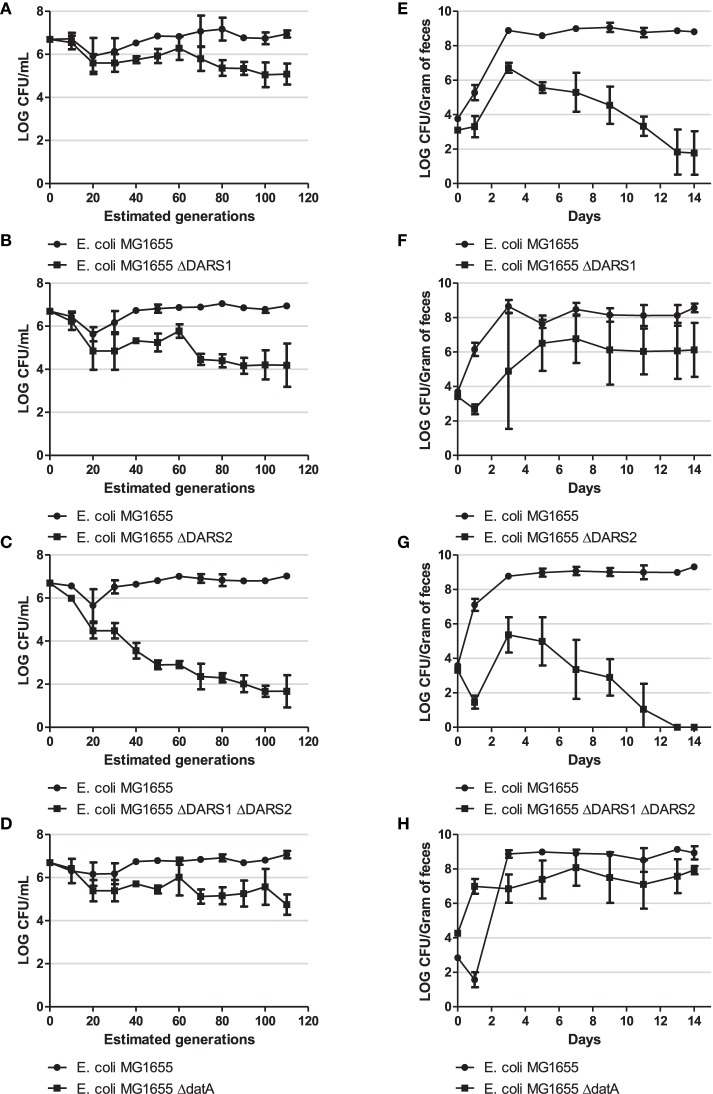
**Competition experiment**. Competition experiment in LB medium **(A–D)** and during colonization of the mouse large intestine **(E–H)** for MG1655 and MG1655 Δ*DARS1* (**A,E**, respectively), MG1655 and MG1655 Δ*DARS2* (**B,F**, respectively), MG1655 and MG1655 Δ*DARS1* Δ*DARS2* (**C,G**, respectively), and MG1655 and MG1655 Δ*datA* (**D,H**, respectively). At the indicated times, samples were homogenized, diluted, and plated as described in Materials and Methods. Bars represent the standard error of the log10 mean number of CFU per mL (competition experiment in LB medium) or CFU per gram of feces (mice experiment).

In order to examine the fitness cost of losing DARS or *datA* activity we performed two different competition experiments: continued growth in LB medium and during mouse colonization, where the streptomycin-treated mouse was chosen as the *in vivo* model. For both competition experiments strains were introduced pairwise at approximately equal numbers (Figure 5). If they have the same fitness they would also be recovered in equal numbers.

During growth in LB the wild-type was more fit than the cells deficient in either *DARS1, DARS2*, both *DARS1* and *DARS2*, or *datA* (Figure 5). The biggest fitness cost resulted from loss of both *DARS1* and *DARS2* (Figure 5C) followed by loss of *DARS2* (Figure 5B) loss of *DARS1* (Figure 5A) which was similar to loss of *datA* (Figure 5D).

The same order of fitness was not observed when evaluated in mice. Following colonization, the number of wild-type *E. coli* increased for about 3 days until stabilizing around 10^9^ cfu per gram of mouse feces (Figures 5E–H). Cells deficient in *DARS1* increased in number to peak at about 10^7^ cfu/gram feces at day 3 followed by a rapid decline in number over the next days to end around 10^2^ cfu/gram feces at day 14 (Figure 5E) suggesting that these cells were rapidly out-competed by wild-type cells. Cells deficient in both *DARS1* and *DARS2* (Figure 5G) were outcompeted at a slightly faster rate than cells deficient in only *DARS1*, suggesting that DARS2 plays a minor role to DARS1 in fitness during mouse colonization. In agreement with this, *DARS2* mutant cells were able to coexist in the mouse along with wild-type cells albeit at a lower number (Figure 5F). Loss of *datA* was similar to the loss of *DARS2*. Following co-infection in mice both wild-type and *datA* cells increased in numbers to level at 10^9^ and 10^7^ cfu per gram feces, respectively, and remained at these levels for the duration of the experiment (Figure 5H). Therefore, *datA* and *DARS2* deficient cells were poor at establishing colonization relative to the wild-type, but once established cells were not outcompeted with time.

On day 14 post-feeding, wild-type and a Δ*DARS1*, Δ*DARS2*, Δ*DARS1* Δ*DARS2*, or Δ*datA* cells (depending on the competition experiment) were isolated from the feces of each mouse for further study. The origin per mass and asynchrony index score were determined for each strain isolated post-infection and found to be similar to the initial strains fed to each mouse [data not shown] showing that secondary mutations were not likely to have been selected during growth in the mouse.

Overall these experiments indicate that different factors determine fitness of cells dependent on growth conditions. During continued growth in LB medium, both promotion and prevention of DnaA^ATP^ to DnaA^ADP^ conversion resulted in a fitness cost. On the other hand, overinitiation resulting from DnaA^ATP^ accumulation in *datA* cells did not seem to affect mouse colonization to the same extent as loss of rejuvenation ability, especially promoted by *DARS1*.

## Discussion

In this study we found conservation in distances from *oriC* to the non-coding regions *DARS1, DARS2, datA*, and *dif* in *E. coli*. *DARS1* and *datA* were always found on the same replichore, while *DARS2* were found on the other replichore. The *oriC, DARS1, DARS2*, and *datA* regions were found to be structurally similar among the tested *E. coli*, with most of the sequence differences found to be in the non-functional spacer regions between key protein binding sites. Cells deficient in *DARS1, DARS2*, or *datA* were viable and had doubling times similar to wild-type. However, replication initiation was perturbed. Cells deficient in *datA* were found to initiate asynchronously, and this could not be counteracted by further deletions of either *DARS1* or *DARS2*. Cells deficient in *DARS1, DARS2, DARS1*, and *DARS2*, or *datA* were found to be less fit than the wild-type in both LB medium and during mouse colonization.

### Conservation of *oriC*

In the chromosomal context initiation of replication can be initiated from a mutant *oriC* without DnaA box R2, R3, R4, or R5, the IBS or the FBS (Weigel et al., 2001), as well as DnaA boxes I1, I2, or I3 (Riber et al., 2009). It is also possible to invert the direction of R4, add 14 bp between DnaA Box R3 and DnaA Box R4, or delete the right half of *oriC* (from position 275 to 352) (Weigel et al., 2001). Although DnaA Box R1 was originally found to be essential (Weigel et al., 2001), a more efficient recombining technique demonstrated that DnaA Box R1 is also dispensable (Kaur et al., 2014). Surprisingly, only deletion of DnaA Box R3, R4, and the right half of *oriC* (from position 275 to 352) was reported to result in slow growth relative to wild-type cells (Weigel et al., 2001). Asynchrony, a sensitive measure for perturbations of the initiation process, was observed with the deletion of the IBS, DnaA Box R2, R4, R5, extending the spacer region between R3 and R4 (Weigel et al., 2001), and the deletion of DnaA Box R1 (Kaur et al., 2014). These studies demonstrate that initiation from *oriC* is very robust and that only major changes in the origin results in loss of function altogether. On the other hand mutant origins fail to compete with their wild-type counterparts as shown by the inability to establish minichromosomes carrying *oriC* mutations in cells with a wild-type chromosomal copy of *oriC* (Weigel et al., 2001). It is conceivable that a similar competition takes place between cells in a population and that even small changes in important regions of *oriC*, that does not affect viability, may result in replication perturbation, loss of fitness and inability to co-exist with wild-type cells and that this explain the high degree of *oriC* conservation observed.

### Chromosomal position of *datA, DARS1*, and *DARS2*

The relative chromosomal locations of the *datA, DARS1*, and *DARS2* regions are conserved among *E. coli* strains. This is somewhat surprising as *E. coli* genomes are highly fluidic, i.e., they frequently mutate, change size, and rearrange. The frequency of genome rearrangement, measured between *rrn* sites, is about 10^3^–10^4^ changes/(generation.genome) (Hill and Gray, 1988). For example, the 1.34 Mb DNA unique to O157:H7 EDL933, is inserted compared to MG1655 in such a way that the chromosomal location of *datA, DARS1*, and *DARS2* relative to *oriC*, remain unchanged. Similarly, the non-essential DNA that was removed from MG1655 to create MSD42 (Pósfai et al., 2006), was also dispersed between regions so that MSD42 has the same relative chromosomal location of *datA, DARS1*, and *DARS2*. There may be at least three reasons for the conserved location of the three regions. First, chromosome asymmetry, i.e., different lengths of the two replication arms leads to slow growth (Hill and Gray, 1988). Second, chromosomal rearrangements resulting in a mixture of different macrodomains have deleterious effects of cell growth (Esnault et al., 2007) which may explain why *datA, DARS1* and *DARS2* regions located within the Ori MD, the Right MD and NS^Left^, respectively (Valens et al., 2004), are always found on the same replichore. Third, the correct chromosomal location of *DARS1, DARS2*, and *datA* may be important for proper function and cell cycle progression (see below). As the activity of these regions in modulating DnaA binding to ATP or ADP is dependent on their copy number the proper distance to *oriC* becomes important for function. The relative copy number of *DARS1, DARS2*, and *datA* (replication-associated gene dosage) decreases with distance from *oriC* (Bremer and Churchward, 1977; Couturier and Rocha, 2006). *datA* was always found close to *oriC*. It is therefore conceivable that, *datA* is duplicated while *oriC* is still sequestered (Kitagawa et al., 1998; Kasho and Katayama, 2013), in all strains. The *datA* site promotes DnaA^ATP^ to DnaA^ADP^ conversion, to prevent re-initiation when the concentration of DnaA^ATP^ is high, i.e., just after sequestration ends and this may provide the evolutionary pressure that has resulted in a conserved chromosomal location. In agreement with this relocation of *datA* to a chromosomal position close to *terC* resulted in high asynchrony in initiation of replication while other positions closer to *oriC* resulted in a near wild-type phenotype (Kitagawa et al., 1998). It is likely that the chromosomal positions of *DARS1* and *DARS2* are also important for cell cycle control as they serve to re-activate the DnaA initiator protein in time for the next initiation. Relocation of DARS sequences has not been experimentally pursued. It is however tempting to speculate that the genomic arrangement of *DARS1* and *DARS2* will ensure that rejuvenation of DnaA^ADP^ to DnaA^ATP^ will be accelerated during later stages of the replication cycle and following duplication of these regions. This rejuvenation is important for increasing the DnaA^ATP^ level for the following round of initiations.

### The *datA, DARS1*, and *DARS2* regions are important for fitness

In the *DARS1* and *DARS2* regions, all DnaA binding boxes as well as spacer distances were conserved among *E. coli* species. Especially the *DARS1* region had a very low mutation frequency which is consistent with reports that all three DnaA boxes along with the region flanking the last DnaA Box (42 bp spanning from base number 198–239) is required for full ADP-releasing activity of *in vitro* (Fujimitsu et al., 2009). Similarly DnaA Box 1 and DnaA Box 2 are crucial for ADP-releasing activity of *DARS2*, while DnaA Box 3 is required for full ADP-releasing activity *in vitro* (Fujimitsu et al., 2009). IBS1–2 and FBS2–3 are required for DnaA^ATP^ regeneration *in vivo* (Kasho et al., 2014). In four *E*. *coli* isolates, FBS-3 has a weaker sequence identity to the Fis consensus sequence than in MG1655. Fis-binding sites are difficult to define due to the lack of an obvious consensus sequence (Finkel and Johnson, 1992), and the effect of the observed weaker identity in FBS-3 is hard to interpret.

*DARS2* was previously reported to be more efficient in rejuvenation of DnaA^ADP^ to DnaA^ATP^ than *DARS1* (Fujimitsu et al., 2009) and both the fitness experiment performed in LB medium (compare Figure 5A and Figure 5B) and the *dnaA* and *mioC* expression studies agreed with this. The situation was reversed in the mouse model where loss of *DARS1* was associated with the biggest fitness cost (compare Figure 5E and Figure 5F). *DARS2* is activated by the binding of both IHF and Fis, whereas no protein factors are required for the function of *DARS1* (Fujimitsu et al., 2009; Kasho et al., 2014). While IHF is abundant in the cell during every growth phase, although the concentration is highest in the stationary phase (Azam and Ishihama, 1999), the concentration of Fis is dependent on the growth phase; i.e., it is highly abundant (10,000–50,000 molecules/cell) in early exponential phase, but decrease to < 100 molecules/cell from late exponential phase to stationary phase. The level of Fis also varies during steady state growth; i.e., it is low during slow growth and high during fast growth (Nilsson et al., 1992; Flåtten and Skarstad, 2013). Limited data are available on the growth of *E. coli* in mouse intestines but overall slow growth was reported with doubling times between 80 and 125 min (Rang et al., 1999). It also seems reasonable that cells under these conditions never reaches exponential growth but grows whenever food becomes available, i.e., with relative short growth phases and frequent entries into stationary phase. Therefore, the bacterial Fis level during intestinal colonization may be significantly lower than during fast exponential growth in rich medium. The relative contribution of *DARS2* to DnaA rejuvenation may therefore be low in the mouse, and explain the bigger fitness cost associated with loss of *DARS1* under these conditions. In agreement with this we only observed a minor further fitness cost associated with deletion of *DARS2* in *DARS1* deficient cells. Such Δ*DARS1* Δ*DARS2* cells rely on *de novo* synthesis of DnaA or the speculated DARS3 to produce DnaA^ATP^ during colonization of a mouse (Kasho et al., 2014). The fitness cost associated with loss of *DARS1* or *DARS2* may readily explain why mutations are rarely observed in these regions, but not the conserved distance to *oriC*. This needs to further elucidated by relocation to other chromosomal positions.

The activity of *datA* is absolutely dependent on DnaA Boxes 2 and 3 along with the IBS (Nozaki et al., 2009; Kasho and Katayama, 2013). Also the spacing between DnaA Box 2 and IBS, as well as the spacing between the IBS and DnaA Box 3 has been shown to be important for *datA* function (Nozaki et al., 2009; Kasho and Katayama, 2013). In accordance with this we found all DnaA boxes as well as the identities to the IHF consensus sequence were conserved. Changes were only observed in the length of the spacer region between DnaA Box 1 and DnaA Box 2 in strain HS and O103:H2 str. 12009 and between the IBS and DnaA Box 3 in strain 536. The effect of the altered spacing is hard to interpret although the latter may lead to a lower efficiency in converting DnaA^ATP^ to DnaA^ADP^ compared to MG1665 (Nozaki et al., 2009). Cells deficient in *datA* only had a 5% decrease in *dnaA* expression correlating with previous reports showing that a *datA* deletion slightly (i.e., 5–10%) increased the DnaA^ATP^ level (Katayama et al., 2001). Loss of *datA* accompanied overinitiation only resulted in modest fitness cost during fast growth in LB and during colonization. The DDAH and RIDA (Hda dependent) pathways both contribute to convert DnaA^ATP^ to DnaA^ADP^ in *E. coli*, but where loss of RIDA is associated with severe overinitiation and inviability unless second site suppressor mutations arise (Riber et al., 2006); loss of DDAH is tolerated. Therefore, DDAH plays a minor role to RIDA and this may explain the limited fitness cost of *datA* cells. The limited fitness cost of losing *datA* relates poorly to the high degree of conservation observed between species. We do not have a good explanation for this observation but it may relate to the DDAH process being important during growth conditions other than those employed by us.

Of interest the Gram-positive bacteria *Bacillus subtilis* and *Streptomyces coelicolor* contain DnaA box clusters close to *oriC* that can repress untimely initiation (Smulczyk-Krawczyszyn et al., 2006; Okumura et al., 2012), i.e., a function similar to that of *datA* in *E. coli*. In addition, several *E. coli* related bacterial species contains *DARS1*-like sequence and *DARS2*-like sequences in a genomic position similar to that of *E. coli* (Fujimitsu et al., 2009; Kasho et al., 2014). These observations indicates that both *datA* and DARSs mechanism, and genomic positions, maybe common to many bacterial species whose genomes contain DnaA box clusters.

## Author contributions

JF and AL planned the experiments. JF performed the experiments. JF, GC, KK, and AL analyzed data. JF and AL wrote the manuscript.

## Funding

This work was supported by grant PIRG05-GA-2009-247241 from the European Union, by grant 09-064250/FNU from the Danish Research Council for Natural sciences, by grant 09-067075 from the Danish Strategic Research Council and by grants from the Lundbeck Foundation and the Novo Nordisk Foundation.

### Conflict of interest statement

The authors declare that the research was conducted in the absence of any commercial or financial relationships that could be construed as a potential conflict of interest.

## References

[B1] Armengod M. E. García-Sogo M. Lambíes E. (1988). Transcriptional organization of the dnaN and recF genes of *Escherichia coli* K-12. J. Biol. Chem. 263, 12109–12114. 2841344

[B2] Azam T. A. Ishihama A. (1999). Twelve species of the nucleoid-associated protein from *Escherichia coli*. Sequence recognition specificity and DNA binding affinity. J. Biol. Chem. 274, 33105–33113. 10.1074/jbc.274.46.33105 10551881

[B3] Bergthorsson U. Ochman H. (1998). Distribution of chromosome length variation in natural isolates of *Escherichia coli*. Mol. Biol. Evol. 15, 6–16. 10.1093/oxfordjournals.molbev.a025847 9491600

[B4] Bigot S. Saleh O. A. Lesterlin C. Pages C. El Karoui M. Dennis C.. (2005). KOPS: DNA motifs that control *E. coli* chromosome segregation by orienting the FtsK translocase. EMBO J. 24, 3770–3780. 10.1038/sj.emboj.7600835 16211009 PMC1276719

[B5] Bogan J. A. Helmstetter C. E. (1997). DNA sequestration and transcription in the oriC region of *Escherichia coli*. Mol. Microbiol. 26, 889–896. 10.1046/j.1365-2958.1997.6221989.x 9426127

[B6] Braun R. E. O'Day K. Wright A. (1985). Autoregulation of the DNA replication gene dnaA in *E. coli* K-12. Cell 40, 159–169. 10.1016/0092-8674(85)90319-8 2981626

[B7] Bremer H. Churchward G. (1977). An examination of the Cooper-Helmstetter theory of DNA replication in bacteria and its underlying assumptions. J. Theor. Biol. 69, 645–654. 10.1016/0022-5193(77)90373-3 607026

[B8] Buss J. Coltharp C. Shtengel G. Yang X. Hess H. Xiao J. (2015). A multi-layered protein network stabilizes the *Escherichia coli* FtsZ-ring and modulates constriction dynamics. PLoS Genet. 11:e1005128. 10.1371/journal.pgen.1005128 25848771 PMC4388696

[B9] Campbell J. L. Kleckner N. (1990). *E. coli* oriC and the dnaA gene promoter are sequestered from dam methyltransferase following the passage of the chromosomal replication fork. Cell 62, 967–979. 10.1016/0092-8674(90)90271-F 1697508

[B10] Campo N. Dias M. J. Daveran-Mingot M. L. Ritzenthaler P. Le Bourgeois P. (2004). Chromosomal constraints in Gram-positive bacteria revealed by artificial inversions. Mol. Microbiol. 51, 511–522. 10.1046/j.1365-2958.2003.03847.x 14756790

[B11] Cherepanov P. P. Wackernagel W. (1995). Gene disruption in *Escherichia coli*: TcR and KmR cassettes with the option of Flp-catalyzed excision of the antibiotic-resistance determinant. Gene 158, 9–14. 10.1016/0378-1119(95)00193-A 7789817

[B12] Couturier E. Rocha E. P. (2006). Replication-associated gene dosage effects shape the genomes of fast-growing bacteria but only for transcription and translation genes. Mol. Microbiol. 59, 1506–1518. 10.1111/j.1365-2958.2006.05046.x 16468991

[B13] Datsenko K. A. Wanner B. L. (2000). One-step inactivation of chromosomal genes in *Escherichia coli* K-12 using PCR products. Proc. Natl. Acad. Sci. U.S.A. 97, 6640–6645. 10.1073/pnas.120163297 10829079 PMC18686

[B14] Eisen J. A. Heidelberg J. F. White O. Salzberg S. L. (2000). Evidence for symmetric chromosomal inversions around the replication origin in bacteria. Genome Biol. 1:RESEARCH0011. 10.1186/gb-2000-1-6-research0011 11178265 PMC16139

[B15] Esnault E. Valens M. Espéli O. Boccard F. (2007). Chromosome structuring limits genome plasticity in *Escherichia coli*. PLoS Genet. 3:e226. 10.1371/journal.pgen.0030226 18085828 PMC2134941

[B16] Espeli O. Borne R. Dupaigne P. Thiel A. Gigant E. Mercier R.. (2012). A MatP-divisome interaction coordinates chromosome segregation with cell division in *E. coli*. EMBO J. 31, 3198–3211. 10.1038/emboj.2012.128 22580828 PMC3400007

[B17] Fekete R. A. Chattoraj D. K. (2005). A cis-acting sequence involved in chromosome segregation in *Escherichia coli*. Mol. Microbiol. 55, 175–183. 10.1111/j.1365-2958.2004.04392.x 15612926

[B18] Finkel S. E. Johnson R. C. (1992). The Fis protein: it's not just for DNA inversion anymore. Mol. Microbiol. 6, 3257–3265. 10.1111/j.1365-2958.1992.tb02193.x 1484481

[B19] Flåtten I. Skarstad K. (2013). The Fis protein has a stimulating role in initiation of replication in *Escherichia coli in vivo*. PLoS ONE 8:e83562. 10.1371/journal.pone.0083562 24358293 PMC3865182

[B20] Fujimitsu K. Senriuchi T. Katayama T. (2009). Specific genomic sequences of *E. coli* promote replicational initiation by directly reactivating ADP-DnaA. Genes Dev. 23, 1221–1233. 10.1101/gad.1775809 19401329 PMC2685538

[B21] Fuller R. S. Funnell B. E. Kornberg A. (1984). The dnaA protein complex with the *E. coli* chromosomal replication origin (oriC) and other DNA sites. Cell 38, 889–900. 10.1016/0092-8674(84)90284-8 6091903

[B22] Grimwade J. E. Ryan V. T. Leonard A. C. (2000). IHF redistributes bound initiator protein, DnaA, on supercoiled oriC of *Escherichia coli*. Mol. Microbiol. 35, 835–844. 10.1046/j.1365-2958.2000.01755.x 10692160

[B23] Groisman E. A. Ochman H. (1997). How Salmonella became a pathogen. Trends Microbiol. 5, 343–349. 10.1016/S0966-842X(97)01099-8 9294889

[B24] Hales L. M. Gumport R. I. Gardner J. F. (1994). Determining the DNA sequence elements required for binding integration host factor to two different target sites. J. Bacteriol. 176, 2999–3006. 8188600 10.1128/jb.176.10.2999-3006.1994 PMC205457

[B25] Hansen E. B. Hansen F. G. Von Meyenburg K. (1982). The nucleotide sequence of the dnaA gene and the first part of the dnaN gene of *Escherichia coli* K-12. Nucleic Acids Res. 10, 7373–7385. 10.1093/nar/10.22.7373 6296774 PMC327010

[B26] Hansen F. G. Christensen B. B. Atlung T. (2007). Sequence characteristics required for cooperative binding and efficient *in vivo* titration of the replication initiator protein DnaA in *E. coli*. J. Mol. Biol. 367, 942–952. 10.1016/j.jmb.2007.01.056 17316685

[B27] Hayashi K. Morooka N. Yamamoto Y. Fujita K. Isono K. Choi S.. (2006). Highly accurate genome sequences of *Escherichia coli* K-12 strains MG1655 and W3110. Mol. Syst. Biol. 2: 2006.0007. 10.1038/msb4100049 16738553 PMC1681481

[B28] Hendrickson H. Lawrence J. G. (2007). Mutational bias suggests that replication termination occurs near the dif site, not at Ter sites. Mol. Microbiol. 64, 42–56. 10.1111/j.1365-2958.2007.05596.x 17376071

[B29] Hill C. W. Gray J. A. (1988). Effects of chromosomal inversion on cell fitness in *Escherichia coli* K-12. Genetics 119, 771–778. 2900793 10.1093/genetics/119.4.771 PMC1203463

[B30] Hill T. M. Henson J. M. Kuempel P. L. (1987). The terminus region of the *Escherichia coli* chromosome contains two separate loci that exhibit polar inhibition of replication. Proc. Natl. Acad. Sci. U.S.A. 84, 1754–1758. 10.1073/pnas.84.7.1754 3550796 PMC304519

[B31] Kaper J. B. Nataro J. P. Mobley H. L. (2004). Pathogenic *Escherichia coli*. Nat. Rev. Microbiol. 2, 123–140. 10.1038/nrmicro818 15040260

[B32] Kasho K. Fujimitsu K. Matoba T. Oshima T. Katayama T. (2014). Timely binding of IHF and Fis to DARS2 regulates ATP-DnaA production and replication initiation. Nucleic Acids Res. 42, 13134–13149. 10.1093/nar/gku1051 25378325 PMC4245941

[B33] Kasho K. Katayama T. (2013). DnaA binding locus datA promotes DnaA-ATP hydrolysis to enable cell cycle-coordinated replication initiation. Proc. Natl. Acad. Sci. U.S.A. 110, 936–941. 10.1073/pnas.1212070110 23277577 PMC3549119

[B34] Katayama T. Fujimitsu K. Ogawa T. (2001). Multiple pathways regulating DnaA function in *Escherichia coli*: distinct roles for DnaA titration by the datA locus and the regulatory inactivation of DnaA. Biochimie 83, 13–17. 10.1016/S0300-9084(00)01206-2 11254969

[B35] Kato J. Katayama T. (2001). Hda, a novel DnaA-related protein, regulates the replication cycle in *Escherichia coli*. EMBO J. 20, 4253–4262. 10.1093/emboj/20.15.4253 11483528 PMC149159

[B36] Kaur G. Vora M. P. Czerwonka C. A. Rozgaja T. A. Grimwade J. E. Leonard A. C. (2014). Building the bacterial orisome: high-affinity DnaA recognition plays a role in setting the conformation of oriC DNA. Mol. Microbiol. 91, 1148–1163. 10.1111/mmi.12525 24443848 PMC3992943

[B37] Kawakami H. Keyamura K. Katayama T. (2005). Formation of an ATP-DnaA-specific initiation complex requires DnaA Arginine 285, a conserved motif in the AAA+ protein family. J. Biol. Chem. 280, 27420–27430. 10.1074/jbc.M502764200 15901724

[B38] Keyamura K. Fujikawa N. Ishida T. Ozaki S. Su'Etsugu M. Fujimitsu K.. (2007). The interaction of DiaA and DnaA regulates the replication cycle in *E. coli* by directly promoting ATP DnaA-specific initiation complexes. Genes Dev. 21, 2083–2099. 10.1101/gad.1561207 17699754 PMC1948862

[B39] Kitagawa R. Ozaki T. Moriya S. Ogawa T. (1998). Negative control of replication initiation by a novel chromosomal locus exhibiting exceptional affinity for *Escherichia coli* DnaA protein. Genes Dev. 12, 3032–3043. 10.1101/gad.12.19.3032 9765205 PMC317192

[B40] Köhler C. D. Dobrindt U. (2011). What defines extraintestinal pathogenic *Escherichia coli*? Int. J. Med. Microbiol. 301, 642–647. 10.1016/j.ijmm.2011.09.006 21982038

[B41] Kurokawa K. Nishida S. Emoto A. Sekimizu K. Katayama T. (1999). Replication cycle-coordinated change of the adenine nucleotide-bound forms of DnaA protein in *Escherichia coli*. EMBO J. 18, 6642–6652. 10.1093/emboj/18.23.6642 10581238 PMC1171727

[B42] Leatham M. P. Stevenson S. J. Gauger E. J. Krogfelt K. A. Lins J. J. Haddock T. L.. (2005). Mouse intestine selects nonmotile flhDC mutants of *Escherichia coli* MG1655 with increased colonizing ability and better utilization of carbon sources. Infect. Immun. 73, 8039–8049. 10.1128/IAI.73.12.8039-8049.2005 16299298 PMC1307065

[B43] Leatham-Jensen M. P. Frimodt-Møller J. Adediran J. Mokszycki M. E. Banner M. E. Caughron J. E.. (2012). The streptomycin-treated mouse intestine selects *Escherichia coli* envZ missense mutants that interact with dense and diverse intestinal microbiota. Infect. Immun. 80, 1716–1727. 10.1128/IAI.06193-11 22392928 PMC3347456

[B44] Leimbach A. Hacker J. Dobrindt U. (2013). *E. coli* as an all-rounder: the thin line between commensalism and pathogenicity. Curr. Top. Microbiol. Immunol. 358, 3–32. 10.1007/978-3-662-45793-1_303 23340801

[B45] Liu S. L. Sanderson K. E. (1995a). The chromosome of Salmonella paratyphi A is inverted by recombination between rrnH and rrnG. J. Bacteriol. 177, 6585–6592. 7592437 10.1128/jb.177.22.6585-6592.1995 PMC177512

[B46] Liu S. L. Sanderson K. E. (1995b). I-CeuI reveals conservation of the genome of independent strains of Salmonella typhimurium. J. Bacteriol. 177, 3355–3357. 7768842 10.1128/jb.177.11.3355-3357.1995 PMC177035

[B47] Løbner-Olesen A. Atlung T. Rasmussen K. V. (1987). Stability and replication control of *Escherichia coli* minichromosomes. J. Bacteriol. 169, 2835–2842. 3294807 10.1128/jb.169.6.2835-2842.1987 PMC212196

[B48] Løbner-Olesen A. Skarstad K. Hansen F. G. von Meyenburg K. Boye E. (1989). The DnaA protein determines the initiation mass of *Escherichia coli* K-12. Cell 57, 881–889. 10.1016/0092-8674(89)90802-7 2541928

[B49] Lu M. Campbell J. L. Boye E. Kleckner N. (1994). SeqA: a negative modulator of replication initiation in *E. coli*. Cell 77, 413–426. 10.1016/0092-8674(94)90156-2 8011018

[B50] Margulies C. Kaguni J. M. (1998). The FIS protein fails to block the binding of DnaA protein to oriC, the *Escherichia coli* chromosomal origin. Nucleic Acids Res. 26, 5170–5175. 10.1093/nar/26.22.5170 9801315 PMC147967

[B51] McGarry K. C. Ryan V. T. Grimwade J. E. Leonard A. C. (2004). Two discriminatory binding sites in the *Escherichia coli* replication origin are required for DNA strand opening by initiator DnaA-ATP. Proc. Natl. Acad. Sci. U.S.A. 101, 2811–2816. 10.1073/pnas.0400340101 14978287 PMC365702

[B52] Medini D. Donati C. Tettelin H. Masignani V. Rappuoli R. (2005). The microbial pan-genome. Curr. Opin. Genet. Dev. 15, 589–594. 10.1016/j.gde.2005.09.006 16185861

[B53] Mercier R. Petit M. A. Schbath S. Robin S. El Karoui M. Boccard F.. (2008). The MatP/matS site-specific system organizes the terminus region of the *E. coli* chromosome into a macrodomain. Cell 135, 475–485. 10.1016/j.cell.2008.08.031 18984159

[B54] Miller C. P. Bohnhoff M. (1963). Changes in the mouse's enteric microflora associated with enhanced susceptibility to salmonella infection following streptomycin treatment. J. Infect. Dis. 113, 59–66. 10.1093/infdis/113.1.59 14044094

[B55] Miller J. (1972). Experiments in Molecular Genetics. Cold Spring Harbor, NY: Cold Spring Harbor Laboratory.

[B56] Niki H. Yamaichi Y. Hiraga S. (2000). Dynamic organization of chromosomal DNA in *Escherichia coli*. Genes Dev. 14, 212–223. 10.1101/gad.14.2.212 10652275 PMC316355

[B57] Nilsson L. Verbeek H. Vijgenboom E. Van Drunen C. Vanet A. Bosch L. (1992). FIS-dependent trans activation of stable RNA operons of *Escherichia coli* under various growth conditions. J. Bacteriol. 174, 921–929. 1732224 10.1128/jb.174.3.921-929.1992 PMC206171

[B58] Nozaki S. Yamada Y. Ogawa T. (2009). Initiator titration complex formed at datA with the aid of IHF regulates replication timing in *Escherichia coli*. Genes Cells 14, 329–341. 10.1111/j.1365-2443.2008.01269.x 19170757

[B59] Ogawa T. Okazaki T. (1994). Cell cycle-dependent transcription from the gid and mioC promoters of *Escherichia coli*. J. Bacteriol. 176, 1609–1615. 8132454 10.1128/jb.176.6.1609-1615.1994 PMC205245

[B60] Okumura H. Yoshimura M. Ueki M. Oshima T. Ogasawara N. Ishikawa S. (2012). Regulation of chromosomal replication initiation by oriC-proximal DnaA-box clusters in Bacillus subtilis. Nucleic Acids Res. 40, 220–234. 10.1093/nar/gkr716 21911367 PMC3245932

[B61] Ozaki S. Katayama T. (2012). Highly organized DnaA-oriC complexes recruit the single-stranded DNA for replication initiation. Nucleic Acids Res. 40, 1648–1665. 10.1093/nar/gkr832 22053082 PMC3287180

[B62] Perna N. T. Plunkett G. III. Burland V. Mau B. Glasner J. D. Rose D. J.. (2001). Genome sequence of enterohaemorrhagic *Escherichia coli* O157:H7. Nature 409, 529–533. 10.1038/35054089 11206551

[B63] Pósfai G. Plunkett G. III. Fehér T. Frisch D. Keil G. M. Umenhoffer K.. (2006). Emergent properties of reduced-genome *Escherichia coli*. Science 312, 1044–1046. 10.1126/science.1126439 16645050

[B64] Rang C. U. Licht T. R. Midtvedt T. Conway P. L. Chao L. Krogfelt K. A.. (1999). Estimation of growth rates of *Escherichia coli* BJ4 in streptomycin-treated and previously germfree mice by *in situ* rRNA hybridization. Clin. Diagn. Lab. Immunol. 6, 434–436. 10225851 10.1128/cdli.6.3.434-436.1999 PMC103738

[B65] Rasko D. A. Rosovitz M. J. Myers G. S. Mongodin E. F. Fricke W. F. Gajer P.. (2008). The pangenome structure of *Escherichia coli*: comparative genomic analysis of *E. coli* commensal and pathogenic isolates. J. Bacteriol. 190, 6881–6893. 10.1128/JB.00619-08 18676672 PMC2566221

[B66] Riber L. Fujimitsu K. Katayama T. Lobner-Olesen A. (2009). Loss of Hda activity stimulates replication initiation from I-box, but not R4 mutant origins in *Escherichia coli*. Mol. Microbiol. 71, 107–122. 10.1111/j.1365-2958.2008.06516.x 19007419

[B67] Riber L. Olsson J. A. Jensen R. B. Skovgaard O. Dasgupta S. Marinus M. G.. (2006). Hda-mediated inactivation of the DnaA protein and dnaA gene autoregulation act in concert to ensure homeostatic maintenance of the *Escherichia coli* chromosome. Genes Dev. 20, 2121–2134. 10.1101/gad.379506 16882985 PMC1536062

[B68] Rozgaja T. A. Grimwade J. E. Iqbal M. Czerwonka C. Vora M. Leonard A. C. (2011). Two oppositely oriented arrays of low-affinity recognition sites in oriC guide progressive binding of DnaA during *Escherichia coli* pre-RC assembly. Mol. Microbiol. 82, 475–488. 10.1111/j.1365-2958.2011.07827.x 21895796 PMC3192301

[B69] Ryan V. T. Grimwade J. E. Camara J. E. Crooke E. Leonard A. C. (2004). *Escherichia coli* prereplication complex assembly is regulated by dynamic interplay among Fis, IHF and DnaA. Mol. Microbiol. 51, 1347–1359. 10.1046/j.1365-2958.2003.03906.x 14982629

[B70] Ryan V. T. Grimwade J. E. Nievera C. J. Leonard A. C. (2002). IHF and HU stimulate assembly of pre-replication complexes at *Escherichia coli* oriC by two different mechanisms. Mol. Microbiol. 46, 113–124. 10.1046/j.1365-2958.2002.03129.x 12366835

[B71] Schaper S. Messer W. (1995). Interaction of the initiator protein DnaA of *Escherichia coli* with its DNA target. J. Biol. Chem. 270, 17622–17626. 10.1074/jbc.270.29.17622 7615570

[B72] Sekimizu K. Bramhill D. Kornberg A. (1987). ATP activates dnaA protein in initiating replication of plasmids bearing the origin of the *E. coli* chromosome. Cell 50, 259–265. 10.1016/0092-8674(87)90221-2 3036372

[B73] Sherratt D. J. Søballe B. Barre F. X. Filipe S. Lau I. Massey T.. (2004). Recombination and chromosome segregation. Philos. Trans. R. Soc. Lond. B Biol. Sci. 359, 61–69. 10.1098/rstb.2003.1365 15065657 PMC1693297

[B74] Skarstad K. Boye E. Steen H. B. (1986). Timing of initiation of chromosome replication in individual *Escherichia coli* cells. EMBO J. 5, 1711–1717. 3527695 10.1002/j.1460-2075.1986.tb04415.x PMC1166998

[B75] Skarstad K. Katayama T. (2013). Regulating DNA replication in bacteria. Cold Spring Harb. Perspect. Biol. 5:a012922. 10.1101/cshperspect.a012922 23471435 PMC3683904

[B76] Smulczyk-Krawczyszyn A. Jakimowicz D. Ruban-Osmialowska B. Zawilak-Pawlik A. Majka J. Chater K.. (2006). Cluster of DnaA boxes involved in regulation of Streptomyces chromosome replication: from *in silico* to *in vivo* studies. J. Bacteriol. 188, 6184–6194. 10.1128/JB.00528-06 16923885 PMC1595370

[B77] Speck C. Messer W. (2001). Mechanism of origin unwinding: sequential binding of DnaA to double- and single-stranded DNA. EMBO J. 20, 1469–1476. 10.1093/emboj/20.6.1469 11250912 PMC145534

[B78] Speck C. Weigel C. Messer W. (1999). ATP- and ADP-dnaA protein, a molecular switch in gene regulation. EMBO J. 18, 6169–6176. 10.1093/emboj/18.21.6169 10545126 PMC1171680

[B79] Valens M. Penaud S. Rossignol M. Cornet F. Boccard F. (2004). Macrodomain organization of the *Escherichia coli* chromosome. EMBO J. 23, 4330–4341. 10.1038/sj.emboj.7600434 15470498 PMC524398

[B80] Weigel C. Messer W. Preiss S. Welzeck M. Morigen Boye, E. (2001). The sequence requirements for a functional *Escherichia coli* replication origin are different for the chromosome and a minichromosome. Mol. Microbiol. 40, 498–507. 10.1046/j.1365-2958.2001.02409.x 11309131

[B81] Wold S. Crooke E. Skarstad K. (1996). The *Escherichia coli* Fis protein prevents initiation of DNA replication from oriC *in vitro*. Nucleic Acids Res. 24, 3527–3532. 10.1093/nar/24.18.3527 8836178 PMC146119

[B82] Yamaichi Y. Niki H. (2004). migS, a cis-acting site that affects bipolar positioning of oriC on the *Escherichia coli* chromosome. EMBO J. 23, 221–233. 10.1038/sj.emboj.7600028 14685268 PMC1271666

