# Supplementary material for: Control regions for chromosome replication are conserved with respect to sequence and location among Escherichia coli strains
Source: Front Microbiol. 2015 Sep 24;6:1011. doi: 10.3389/fmicb.2015.01011 (PMC4585315; doi:10.3389/fmicb.2015.01011)
Supplement: Supplementary file 1 [file Presentation2.PDF]

Supplementary Material

Control regions for chromosome replication are conserved with respect to both sequence and location among *Escherichia coli* strains

Jakob Frimodt-Møller[1,2], Godefroid Charbon[1], Karen A. Krogfelt[2], Anders Løbner-Olesen*[1]

[1] Dept. of Biology, Functional Genomics, University of Copenhagen, Copenhagen, Denmark.

[2] Dept. of Microbiology and Infection Control, Statens Serum Institut, Copenhagen, Denmark

*** Correspondence:** Anders Løbner-Olesen, Dept. of Biology, Functional Genomics, University of Copenhagen, Copenhagen, 2200, Denmark

lobner@bio.ku.dk

1 Supplementary Figures

Supplementary Figure S1. Comparison of heterogeneous *oriC* sequences for the 13-mer termed R.

DUE region 13-mer R in *oriC*

E. coli MG1655	G A T C T C T T A T T A G
E. coli O157:H7 EDL933	T A T C T C T T A T T A G
E. coli O157:H7 str. Sakai	T A T C T C T T A T T A G
E. coli O157:H7 str. EC4115	T A T C T C T T A T T A G
E. coli O157:H7 str. TW14359	T A T C T C T T A T T A G
E. coli Xuzhou21	T A T C T C T T A T T A G
E. coli ED1a	G A T C T C T T A T T A A
***E. coli* CONSENSUS SEQUENCE**	**K A T C T C T T A T T A R**

K = G/T; R = A/G

Identical nucleotides are indicated in blue and nucleotides different from the MG1655 sequence is given in red. The R 13-mer consensus sequence for the 59 tested *E. coli* is given.

Supplementary Figure S2. Comparison of single stranded DnaA^{ATP} boxes and the AT-region.

Single-stranded DnaA-ATP boxes (SSDA)

SSDA Box 1

E. coli MG1655	G G A T C G
E. coli ED1a	A G A T C G

SSDA Box 2

E. coli MG1655	T G A T C T
E. coli O157:H7 EDL933	T T A T C T
E. coli O157:H7 str. Sakai	T T A T C T
E. coli O157:H7 str. EC4115	T T A T C T
E. coli O157:H7 str. TW14359	T T A T C T
E. coli Xuzhou21	T T A T C T

SSDA Box 3 (CONSERVED)

E. coli MG1655	A G A T C T

SSDA Box 4 (CONSERVED)

E. coli MG1655	A G A T C T

SSDA Box 5

E. coli MG1655	T G A T C C
E. coli APEC O78	T G A T C T

SSDA Box 6 (CONSERVED)

E. coli MG1655	G G A T C C

AT (CONSERVED)

E. coli MG1655	T A T T A A A A A G A A

Identical nucleotides are indicated in blue and nucleotides different from MG1655 sequence is given in red.

Supplementary Figure S3. Comparison of heterogeneous *oriC* sequences for the DnaA Box I1.

DnaA Box I1 in *oriC*

E. coli MG1655	**T T A T A C G G T**
R-BOX CONSENSUS SEQUENCE	**T T W T N C A C A**
E. coli 0127:H6 E2348/69	**T T A T A C T G T**
E. coli UM146	**T T A T A C G G C**
E. coli S88	**T T A T A C G G C**
E. coli IHE3034	**T T A T A C G G C**
E. coli PMV-1	**T T A T A C G G C**
E. coli UTI89	**T T A T A C G G C**
***E. coli* CONSENSUS SEQUENCE**	**T T A T A C K G Y**

W = A/T, N = Any nucleotide; K = G/T; Y = C/T

DnaA Box I1 sequence is given for MG1655 along with the DnaA R-box consensus sequence. Nucleotides are colored blue if they have an unchanged identity to the consensus sequence, colored red if they have a diminished identity to the consensus sequence, and colored green if they have a better resemble to the consensus sequence compared to MG1655. The DnaA Box I1 consensus sequence for the 59 tested *E. coli* is given.

Supplementary Figure S4. Comparison of heterogeneous *oriC* sequences for the DnaA Box I2.

DnaA Box I2 in *oriC*

E. coli MG1655	**C T G A T C C C A**
R-BOX CONSENSUS SEQUENCE*	**T T W T N C A C A**
E. coli ED1a	**C T A A T C C C A**
***E. coli* CONSENSUS SEQUENCE**	**C T R A T C C C G**

W = A/T; N = Any nucleotide; R = A/G

DnaA Box I2 sequence is given for MG1655 along with the DnaA R-box consensus sequence. Nucleotides are colored blue if they have an unchanged identity to the consensus sequence, colored red if they have a diminished identity to the consensus sequence, and colored green if they have a better resemble to the consensus sequence compared to MG1655. The DnaA Box I2 consensus sequence for the 59 tested *E. coli* is given.

Supplementary Figure S5. Comparison of heterogeneous *oriC* sequences for the DnaA Box C3 and DnaA Box R3.

DnaA Box C3 in *oriC*

E. coli MG1655	T T G T T C T T T
R-BOX CONSENSUS SEQUENCE	T T W T N C A C A
E. coli B str. REL606	T T G T T C C T T
E. coli W	T T G T T C C T T
E. coli P12b	T T G T T C C T T
E. coli ATCC 8739	T T G T T C C T T
E. coli O26:H11 str. 11368	T T G T T C C T T
E. coli O103:H2 str. 12009 DNA	T T G T T C C T T
E. coli HS	T T G T T C C T T
E. coli IAI1	T T G T T C C T T
E. coli APEC O78	T T G T T C C T T
E. coli UMNK88	T T G T T C C T T
***E. coli* CONSENSUS SEQUENCE**	**T T G T T C Y T T**

DnaA Box R3 in *oriC*

E. coli MG1655	T T A T C C A A A
R-BOX CONSENSUS SEQUENCE	T T W T N C A C A
E. coli B str. REL606	T T A T C C A A G
E. coli W	T T A T C C A A G
E. coli P12b	T T A T C C A A G
E. coli ATCC 8739	T T A T C C A A G
E. coli O26:H11 str. 11368	T T A T C C A A G
E. coli O103:H2 str. 12009 DNA	T T A T C C A A G
E. coli HS	T T A T C C A A G
E. coli IAI1	T T A T C C A A G
E. coli APEC O78	T T A T C C A A G
E. coli UMNK88	T T A T C C A A G
***E. coli* CONSENSUS SEQUENCE**	**T T A T C C A A R**

W = A/T; n = Any nucleotide; Y = C/T; R = A/G

DnaA Box C3/R3 sequence is given for MG1655 along with the DnaA R-box consensus sequence. Nucleotides are colored blue if they have an unchanged identity to the consensus sequence, colored red if they have a diminished identity to the consensus sequence, and colored green if they have a better resemble to the consensus sequence compared to MG1655. The DnaA Box C3 and R3 consensus sequence for the 59 tested *E. coli* is given.

Supplementary Figure S6. Comparison of heterogeneous *oriC* sequences for the DnaA Box R4.

DnaA Box R4 in *oriC*

E. coli MG1655	**T T A T C C A C A**
R-BOX CONSENSUS SEQUENCE	**T T W T N C A C A**
E. coli 0127:H6 E2348/69	**T T A C C C A C A**
***E. coli* CONSENSUS SEQUENCE**	**T T A Y C C A C A**

W = A/T, n = Any nucleotide; Y = C/T

DnaA Box R4 sequence is given for MG1655 along with the DnaA R-box consensus sequence. Nucleotides are colored blue if they have an unchanged identity to the consensus sequence, colored red if they have a diminished identity to the consensus sequence, and colored green if they have a better resemble to the consensus sequence compared to MG1655. The DnaA Box R4 consensus sequence for the 59 tested *E. coli* is given.

Supplementary Figure S7. Comparison of heterogeneous *oriC* sequences for the IHF Binding Site.

IHF Binding Site in *oriC*

E. coli MG1655	**G A T C A A C A A C C T G**
CONSENSUS SEQUENCE	**W A T C A A N N N N T T R**
E. coli O26:H11 str. 11368	**G A T C A A C A A C C G G**
E. coli O145:H28 str. RM12761	**A A T C A A C A A C C T G**
E. coli O145:H28 str. RM13516	**A A T C A A C A A C C T G**
***E. coli* CONSENSUS SEQUENCE**	**R A T C A A C A A C C K G**

*W = A/T; N = Any nucleotide; R = A/G; K = T/G

IHF binding site sequence is given for MG1655 along with the IHF consensus sequence. Nucleotides are colored blue if they have an unchanged identity to the consensus sequence, colored red if they have a diminished identity to the consensus sequence, and colored green if they have a better resemble to the consensus sequence compared to MG1655. The IHF consensus sequence for the 59 tested *E. coli* is given.

Supplementary Figure S8. Comparison of heterogeneous DASR2 sequences for the Fis Binding Site 2.

Fis Binding Site 2 in DARS2

E. coli MG1655	G G C G A A A G A T C A A C C
CONSENSUS SEQUENCE	G N N Y A N N N N N T R N N C
E. coli B str. REL606	G A C G A A A G A T C A A C C
E. coli HS	G A C G A A A G A T C A A C C
E. coli strain ST2747	G G C G A A A G A T C A G C C
***E. coli* CONSENSUS SEQUENCE**	G R C G A A A G A T C A R C C

Y = C/T; R = A/G; N = Any nucleotide

Fis binding site 2 sequence is given for MG1655 along with the Fis consensus sequence. Nucleotides are colored blue if they have an unchanged identity to the consensus sequence, colored red if they have a diminished identity to the consensus sequence, and colored green if they have a better resemble to the consensus sequence compared to MG1655. The Fis binding site 2 consensus sequence for the 59 tested *E. coli* is given.

Supplementary Figure S9. Comparison of heterogeneous DASR2 sequences for the Fis Binding Site 3.

Fis Binding Site 3 in DARS2

E. coli MG1655	**G A T C A A C C A A T G C C G**
CONSENSUS SEQUENCE	**G N N Y A N N N N N T R N N C**
E. coli SE15	G A T C A A C C A A C G C C G
E. coli NA114	G A T C A A C C A A C G C C G
E. coli O25b:H4-ST131 str. EC958	G A T C A A C C A A C G C C G
E. coli JJ1886	G A T C A A C C A A C G C C G
E. coli 042	G A T C A A C C A A T G C G G
E. coli IAI39	G A T C A A C C A A T G C G G
E. coli O7:K1 str. CE10	G A T C A A C C A A T G C G G
E. coli UMN026	G A T C A A C C A A T G C G G
E. coli strain ST2747	G A T C A G C C A A T G C C G
***E. coli* CONSENSUS SEQUENCE**	**G A T C A R C C A A Y G C S G**

Y = C/T; R = A/G; N = Any nucleotide; S = C/G

Fis binding site 3 sequence is given for MG1655 along with the Fis consensus sequence. Nucleotides are colored blue if they have an unchanged identity to the consensus sequence, colored red if they have a diminished identity to the consensus sequence, and colored green if they have a better resemble to the consensus sequence compared to MG1655. The Fis binding site 3 consensus sequence for the 59 tested *E. coli* is given.

Supplementary Figure S10. Comparison of heterogeneous DARS2 sequences for the IHF Binding Site 2.

IHF Binding Site 2 in DARS2

E. coli MG1655	**A G T C A A T G C A T T A**
CONSENSUS SEQUENCE	**W A T C A A N N N N T T A**
E. coli SE15	**A G T C A A T G G A T T A**
E. coli NA114	**A G T C A A T G G A T T A**
E. coli O25b:H4-ST131 str. EC958	**A G T C A A T G G A T T A**
E. coli JJ1886	**A G T C A A T G G A T T A**
***E. coli* CONSENSUS SEQUENCE**	**A G T C A A T G S A T T A**

W = A/T; N = Any nucleotide; S = C/G

IHF binding site 2 sequence is given for MG1655 along with the IHF consensus sequence. Nucleotides are colored blue if they have an unchanged identity to the consensus sequence, colored red if they have a diminished identity to the consensus sequence, and colored green if they have a better resemble to the consensus sequence compared to MG1655. The IHF binding site 2 consensus sequence for the 59 tested *E. coli* is given.

Supplementary Figure S11. Comparison of heterogeneous *datA* sequences for the IHF Binding Site.

IHF Binding Site in *datA*

E. coli MG1655	T A T C A A T A G G T T A
CONSENSUS SEQUENCE*	**W A T C A A N N N N T T A**
E. coli W	T A T C A A T A A G T T A
E. coli O26:H11 str. 11368	T A T C A A T A A G T T A
E. coli O111:H- str. 11128	T A T C A A T A A G T T A
E. coli 042	T A T C A A T A A G T T A
E. coli O7:K1 str. CE10	T A T C A A T A A G T T A
E. coli IAI39	T A T C A A T A A G T T A
E. coli O104:H4 str. 2009EL-2050	T A T C A A T A T G T T A
E. coli O104:H4 str. 2009EL-2071	T A T C A A T A T G T T A
E. coli O104:H4 str. 2011C-3493	T A T C A A T A T G T T A
E. coli 55989	T A T C A A T A T G T T A
E. coli UMN026	T A T C A A T A G A T T A
E. coli ST2747	T A T C A A T A G A T T A
***E. coli* CONSENSUS SEQUENCE**	**T A T C A A T A D R T T A**

W = A/T; N = Any nucleotide; R = A/G; D = A/G/T

IHF binding site sequence is given for MG1655 along with the IHF consensus sequence. Nucleotides are colored blue if they have an unchanged identity to the consensus sequence, colored red if they have a diminished identity to the consensus sequence, and colored green if they have a better resemble to the consensus sequence compared to MG1655. The IHF binding site consensus sequence for the 59 tested *E. coli* is given.

Supplementary Figure S12. Comparison of the minimal *oriC* sequence.

Minimal *oriC* sequence from MG1655 aligned with the remaining 58 *E. coli* isolates. Nucleotides that differs from the MG1655 sequence is colored in red. Note that the DnaA box R3 overlaps with DnaA box C3 and C2 in *oriC*. IHF, IHF-binding site; FBS, Fis-binding site.

Supplementary Figure S13. Comparison of the *datA* sequence.

The *datA* sequence from MG1655 aligned with the remaining 58 *E. coli* isolates. Nucleotides that differs from the MG1655 sequence is colored in red. IHF, IHF-binding site.

Supplementary Figure S14. Comparison of the *DARS1* sequence.

The DARS1 sequence from MG1655 aligned with the remaining 58 *E. coli* isolates. Nucleotides that differs from the MG1655 sequence is colored in red.

Supplementary Figure S15. Comparison of the *DARS2* sequence.

The DARS2 sequence from MG1655 aligned with the remaining 58 *E. coli* isolates. Nucleotides that differs from the *E. coli* MG1655 sequence is colored in red. IHF, IHF-binding site; FBS, Fis-binding site.

Supplementary Figure S12

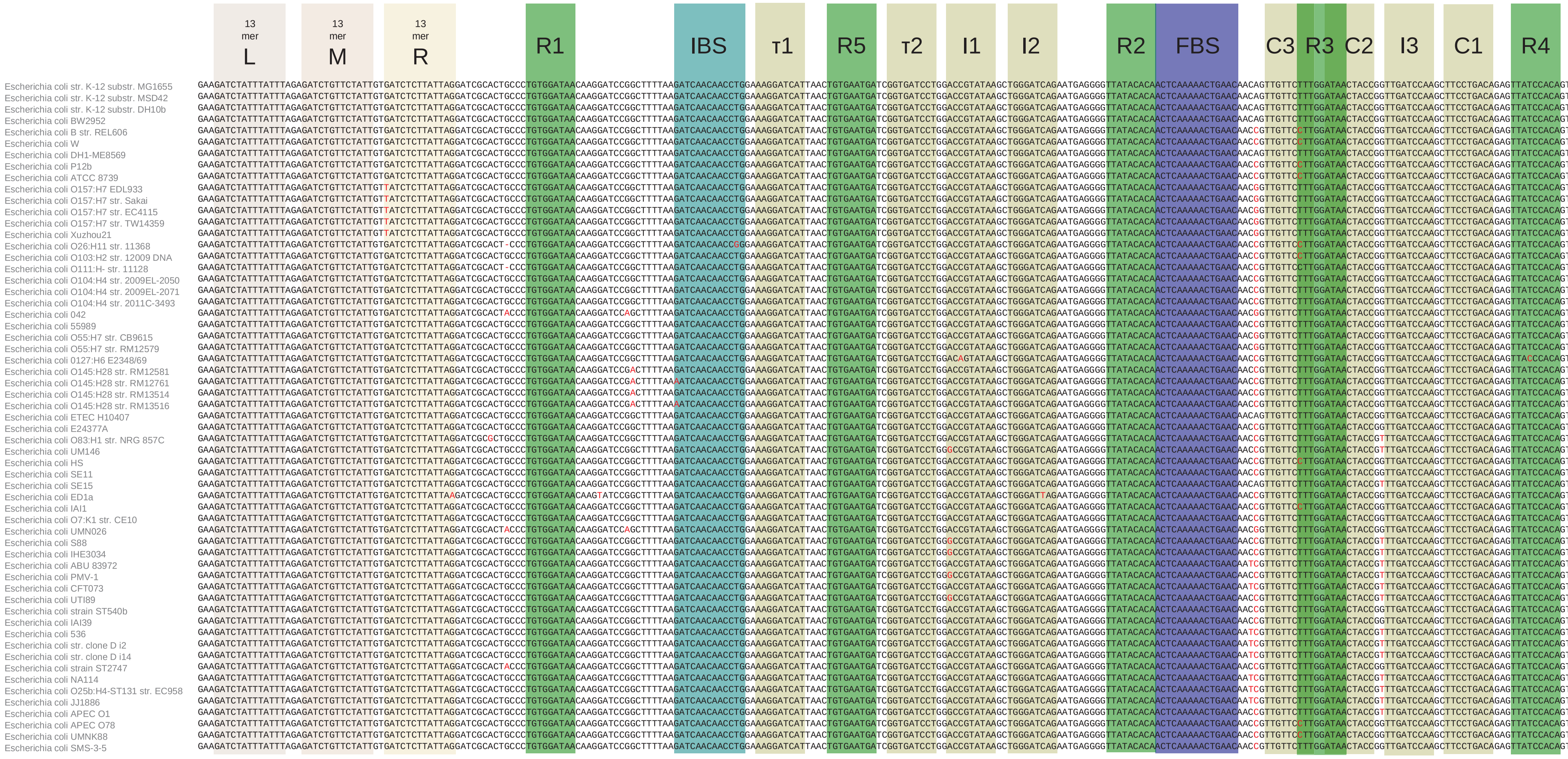

Supplementary Figure S13

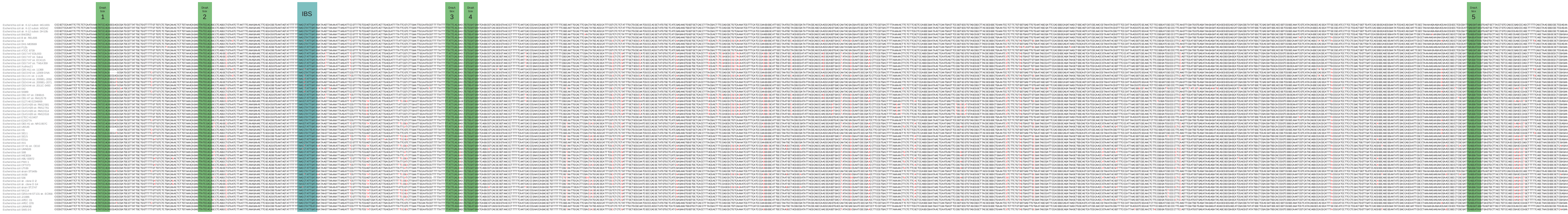

Supplementary Figure S14

DnaA box 1 | DnaA box 2 | DnaA box 3

Escherichia coli str. K-12 substr. MG1655	GGGGATAGGGGCTGGAGACAGTTATCCACTATTCCTGTGGATAACCATGTGTATTAGAGTTAGAAAACACGAGGCAAGCGAGAGAATACGCGGCTTGCACG
Escherichia coli str. K-12 substr. MSD42	GGGGATAGGGGCTGGAGACAGTTATCCACTATTCCTGTGGATAACCATGTGTATTAGAGTTAGAAAACACGAGGCAAGCGAGAGAATACGCGGCTTGCACG
Escherichia coli str. K-12 substr. DH10b	GGGGATAGGGGCTGGAGACAGTTATCCACTATTCCTGTGGATAACCATGTGTATTAGAGTTAGAAAACACGAGGCAAGCGAGAGAATACGCGGCTTGCACG
Escherichia coli BW2952	GGGGATAGGGGCTGGAGACAGTTATCCACTATTCCTGTGGATAACCATGTGTATTAGAGTTAGAAAACACGAGGCAAGCGAGAGAATACGCGGCTTGCACG
Escherichia coli B str. REL606	GGGGATAGGGGCTGGAGACAGTTATCCACTATTCCTGTGGATAACCATGTGTATTAGAGTTAGAAAACACGAGGCAAGCGAGAGAATACGCGGCTTGCACG
Escherichia coli W	GGGGATAGGGGCTGGAGACAGTTATCCACTATTCCTGTGGATAACCATGTGTATTAGAGTTAGAAAACACGAGGCAAGCGAGAGAATACGCGGCTTGCACG
Escherichia coli DH1-ME8569	GGGGATAGGGGCTGGAGACAGTTATCCACTATTCCTGTGGATAACCATGTGTATTAGAGTTAGAAAACACGAGGCAAGCGAGAGAATACGCGGCTTGCACG
Escherichia coli P12b	GGGGATAGGGGCTGGAGACAGTTATCCACTATTCCTGTGGATAACCATGTGTATTAGAGTTAGAAAACACGAGGCAAGCGAGAGAATACGCGGCTTGCACG
Escherichia coli ATCC 8739	GGGGATAGGGGCTGGAGACAGTTATCCACTATTCCTGTGGATAACCATGTGTATTAGAGTTAGAAAACACGAGGCAAGCGAGAGAATACGCGGCTTGCACG
Escherichia coli O157:H7 EDL933	GGGGATAGGGGCTGCAGACAGTTATCCACTATTCCTGTGGATAACCATGTGTATTAGAGTTAGAAAACACGAGGCAAGCGAGAGAATACGCGGCTTGCACG
Escherichia coli O157:H7 str. Sakai	GGGGATAGGGGCTGCAGACAGTTATCCACTATTCCTGTGGATAACCATGTGTATTAGAGTTAGAAAACACGAGGCAAGCGAGAGAATACGCGGCTTGCACG
Escherichia coli O157:H7 str. EC4115	GGGGATAGGGGCTGCAGACAGTTATCCACTATTCCTGTGGATAACCATGTGTATTAGAGTTAGAAAACACGAGGCAAGCGAGAGAATACGCGGCTTGCACG
Escherichia coli O157:H7 str. TW14359	GGGGATAGGGGCTGCAGACAGTTATCCACTATTCCTGTGGATAACCATGTGTATTAGAGTTAGAAAACACGAGGCAAGCGAGAGAATACGCGGCTTGCACG
Escherichia coli Xuzhou21	GGGGATAGGGGCTGCAGACAGTTATCCACTATTCCTGTGGATAACCATGTGTATTAGAGTTAGAAAACACGAGGCAAGCGAGAGAATACGCGGCTTGCACG
Escherichia coli O26:H11 str. 11368	GGGGATAGGGGCTGGAGACAGTTATCCACTATTCCTGTGGATAACCATGTGTATTAGAGTTAGAAAACACGAGGCAAGCGAGAGAATACGCGGCTTGCACG
Escherichia coli O103:H2 str. 12009 DNA	GGGGATAGGGGCTGGAGACAGTTATCCACTATTCCTGTGGATAACCATGTGTATTAGAGTTAGAAAACACGAGGCAAGCGAGAGAATACGCGGCTTGCACG
Escherichia coli O111:H- str. 11128	GGGGATAGGGGCTGGAGACAGTTATCCACTATTCCTGTGGATAACCATGTGTATTAGAGTTAGAAAACACGAGGCAAGCGAGAGAATACGCGGCTTGCACG
Escherichia coli O104:H4 str. 2009EL-2050	GGGGATAGGGGCTGGAGACAGTTATCCACTATTCCTGTGGATAACCATGTGTATTAGAGTTAGAAAACACGAGGCAAGCGAGAGAATACGCGGCTTGCACG
Escherichia coli O104:H4 str. 2009EL-2071	GGGGATAGGGGCTGGAGACAGTTATCCACTATTCCTGTGGATAACCATGTGTATTAGAGTTAGAAAACACGAGGCAAGCGAGAGAATACGCGGCTTGCACG
Escherichia coli O104:H4 str. 2011C-3493	GGGGATAGGGGCTGGAGACAGTTATCCACTATTCCTGTGGATAACCATGTGTATTAGAGTTAGAAAACACGAGGCAAGCGAGAGAATACGCGGCTTGCACG
Escherichia coli 042	GGGGATAGGGGCTGCAGACAGTTATCCACTATTCCTGTGGATAACCATGTGTATTAGAGTTAGAAAACACGAGGCAAGCGAGAGAATACGCGGCTTGCACG
Escherichia coli 55989	GGGGATAGGGGCTGGAGACAGTTATCCACTATTCCTGTGGATAACCATGTGTATTAGAGTTAGAAAACACGAGGCAAGCGAGAGAATACGCGGCTTGCACG
Escherichia coli O55:H7 str. CB9615	GGGGATAGGGGCTGCAGACAGTTATCCACTATTCCTGTGGATAACCATGTGTATTAGAGTTAGAAAACACGAGGCAAGCGAGAGAATACGCGGCTTGCACG
Escherichia coli O55:H7 str. RM12579	GGGGATAGGGGCTGCAGACAGTTATCCACTATTCCTGTGGATAACCATGTGTATTAGAGTTAGAAAACACGAGGCAAGCGAGAGAATACGCGGCTTGCACG
Escherichia coli 0127:H6 E2348/69	GGGGATAGGGGCTGCAGACAGTTATCCACTATTCCTGTGGATAACCATGTGTATTAGAGTTAGAAAACACGAGGCAAGCGAGAGAATACGCGGCTTGCACG
Escherichia coli O145:H28 str. RM12581	GGGGATAGGGGCTGCAGACAGTTATCCACTATTCCTGTGGATAACCATGTGTATTAGAGTTAGAAAACACGAGGCAAGCGAGAGAATACGCGGCTTGCACG
Escherichia coli O145:H28 str. RM12761	GGGGATAGGGGCTGCAGACAGTTATCCACTATTCCTGTGGATAACCATGTGTATTAGAGTTAGAAAACACGAGGCAAGCGAGAGAATACGCGGCTTGCACG
Escherichia coli O145:H28 str. RM13514	GGGGATAGGGGCTGCAGACAGTTATCCACTATTCCTGTGGATAACCATGTGTATTAGAGTTAGAAAACACGAGGCAAGCGAGAGAATACGCGGCTTGCACG
Escherichia coli O145:H28 str. RM13516	GGGGATAGGGGCTGCAGACAGTTATCCACTATTCCTGTGGATAACCATGTGTATTAGAGTTAGAAAACACGAGGCAAGCGAGAGAATACGCGGCTTGCACG
Escherichia coli ETEC H10407	GGGGATAGGGGCTGGAGACAGTTATCCACTATTCCTGTGGATAACCATGTGTATTAGAGTTAGAAAACACGAGGCAAGCGAGAGAATACGCGGCTTGCACG
Escherichia coli E24377A	GGGGATAGGGGCTGGAGACAGTTATCCACTATTCCTGTGGATAACCATGTGTATTAGAGTTAGAAAACACGAGGCAAGCGAGAGAATACGCGGCTTGCACG
Escherichia coli O83:H1 str. NRG 857C	GGGGATAGGGGCTGCAGACAGTTATCCACTATTCCTGTGGATAACCATGTGTATTAGAGTTAGAAAACACGAGGCAAGCGAGAGAATACGCGGCTTGCACG
Escherichia coli UM146	GGGGATAGGGGCTGCAGACAGTTATCCACTATTCCTGTGGATAACCATGTGTATTAGAGTTAGAAAACACGAGGCAAGCGAGAGAATACGCGGCTTGCACG
Escherichia coli HS	GGGGATAGGGGCTGGAGACAGTTATCCACTATTCCTGTGGATAACCATGTGTATTAGAGTTAGAAAACACGAGGCAAGCGAGAGAATACGCGGCTTGCACG
Escherichia coli SE11	GGGGATAGGGGCTGGAGACAGTTATCCACTATTCCTGTGGATAACCATGTGTATTAGAGTTAGAAAACACGAGGCAAGCGAGAGAATACGCGGCTTGCACG
Escherichia coli SE15	GGGGATAGGGGCTGCAGACAGTTATCCACTATTCCTGTGGATAACCATGTGTATTAGAGTTAGAAAACACGAGGCAAGCGAGAGAATACGCGGCTTGCACG
Escherichia coli ED1a	GGGGATAGGGGCTGCAGACAGTTATCCACTATTCCTGTGGATAACCATGTGTATTAGAGTTAGAAAACACGAGGCAAGCGAGAGAATACGCGGCTTGCACG
Escherichia coli IAI1	GGGGATAGGGGCTGGAGACAGTTATCCACTATTCCTGTGGATAACCATGTGTATTAGAGTTAGAAAACACGAGGCAAGCGAGAGAATACGCGGCTTGCACG
Escherichia coli O7:K1 str. CE10	GGGGATAGGGGCTGCAGACAGTTATCCACTATTCCTGTGGATAACCATGTGTATTAGAGTTAGAAAACACGAGGCAAGCGAGAGAATACGCGGCTTGCACG
Escherichia coli UMN026	GGGGATAGGGGCTGCAGACAGTTATCCACTATTCCTGTGGATAACCATGTGTATTAGAGTTAGAAAACACGAGGCAAGCGAGAGAATACGCGGCTTGCACG
Escherichia coli S88	GGGGATAGGGGCTGCAGACAGTTATCCACTATTCCTGTGGATAACCATGTGTATTAGAGTTAGAAAACACGAGGCAAGCGAGAGAATACGCGGCTTGCACG
Escherichia coli IHE3034	GGGGATAGGGGCTGCAGACAGTTATCCACTATTCCTGTGGATAACCATGTGTATTAGAGTTAGAAAACACGAGGCAAGCGAGAGAATACGCGGCTTGCACG
Escherichia coli ABU 83972	GGGGATAGGGGCTGCAGACAGTTATCCACTATTCCTGTGGATAACCATGTGTATTAGAGTTAGAAAACACGAGGCAAGCGAGAGAATACGCGGCTTGCACG
Escherichia coli PMV-1	GGGGATAGGGGCTGCAGACAGTTATCCACTATTCCTGTGGATAACCATGTGTATTAGAGTTAGAAAACACGAGGCAAGCGAGAGAATACGCGGCTTGCACG
Escherichia coli CFT073	GGGGATAGGGGCTGCAGACAGTTATCCACTATTCCTGTGGATAACCATGTGTATTAGAGTTAGAAAACACGAGGCAAGCGAGAGAATACGCGGCTTGCACG
Escherichia coli UTI89	GGGGATAGGGGCTGCAGACAGTTATCCACTATTCCTGTGGATAACCATGTGTATTAGAGTTAGAAAACACGAGGCAAGCGAGAGAATACGCGGCTTGCACG
Escherichia coli strain ST540b	GGGGATAGGGGCTGGAGACAGTTATCCACTATTCCTGTGGATAACCATGTGTATTAGAGTTAGAAAACACGAGGCAAGCGAGAGAATACGCGGCTTGCACG
Escherichia coli IAI39	GGGGATAGGGGCTGCAGACAGTTATCCACTATTCCTGTGGATAACCATGTGTATTAGAGTTAGAAAACACGAGGCAAGCGAGAGAATACGCGGCTTGCACG
Escherichia coli 536	GGGGATAGGGGCTGCAGACAGTTATCCACTATTCCTGTGGATAACCATGTGTATTAGAGTTAGAAAACACGAGGCAAGCGAGAGAATACGCGGCTTGCACG
Escherichia coli str. clone D i2	GGGGATAGGGGCTGCAGACAGTTATCCACTATTCCTGTGGATAACCATGTGTATTAGAGTTAGAAAACACGAGGCAAGCGAGAGAATACGCGGCTTGCACG
Escherichia coli str. clone D i14	GGGGATAGGGGCTGCAGACAGTTATCCACTATTCCTGTGGATAACCATGTGTATTAGAGTTAGAAAACACGAGGCAAGCGAGAGAATACGCGGCTTGCACG
Escherichia coli strain ST2747	GGGGATAGGGGCTGCAGACAGTTATCCACTATTCCTGTGGATAACCATGTGTATTAGAGTTAGAAAACACGAGGCAAGCGAGAGAATACGCGGCTTGCACG
Escherichia coli NA114	GGGGATAGGGGCTGCAGACAGTTATCCACTATTCCTGTGGATAACCATGTGTATTAGAGTTAGAAAACACGAGGCAAGCGAGAGAATACGCGGCTTGCACG
Escherichia coli O25b:H4-ST131 str. EC958	GGGGATAGGGGCTGCAGACAGTTATCCACTATTCCTGTGGATAACCATGTGTATTAGAGTTAGAAAACACGAGGCAAGCGAGAGAATACGCGGCTTGCACG
Escherichia coli JJ1886	GGGGATAGGGGCTGCAGACAGTTATCCACTATTCCTGTGGATAACCATGTGTATTAGAGTTAGAAAACACGAGGCAAGCGAGAGAATACGCGGCTTGCACG
Escherichia coli APEC O1	GGGGATAGGGGCTGCAGACAGTTATCCACTATTCCTGTGGATAACCATGTGTATTAGAGTTAGAAAACACGAGGCAAGCGAGAGAATACGCGGCTTGCACG
Escherichia coli APEC O78	GGGGATAGGGGCTGGAGACAGTTATCCACTATTCCTGTGGATAACCATGTGTATTAGAGTTAGAAAACACGAGGCAAGCGAGAGAATACGCGGCTTGCACG
Escherichia coli UMNK88	GGGGATAGGGGCTGCAGACAGTTATCCACTATTCCTGTGGATAACCATGTGTATTAGAGTTAGAAAACACGAGGCAAGCGAGAGAATACGCGGCTTGCACG
Escherichia coli SMS-3-5	GGGGATAGGGGCTGCAGACAGTTATCCACTATTCCTGTGGATAACCATGTGTATTAGAGTTAGAAAACACGAGGCAAGCGAGAGAATACGCGGCTTGCACG

Supplementary Figure S15

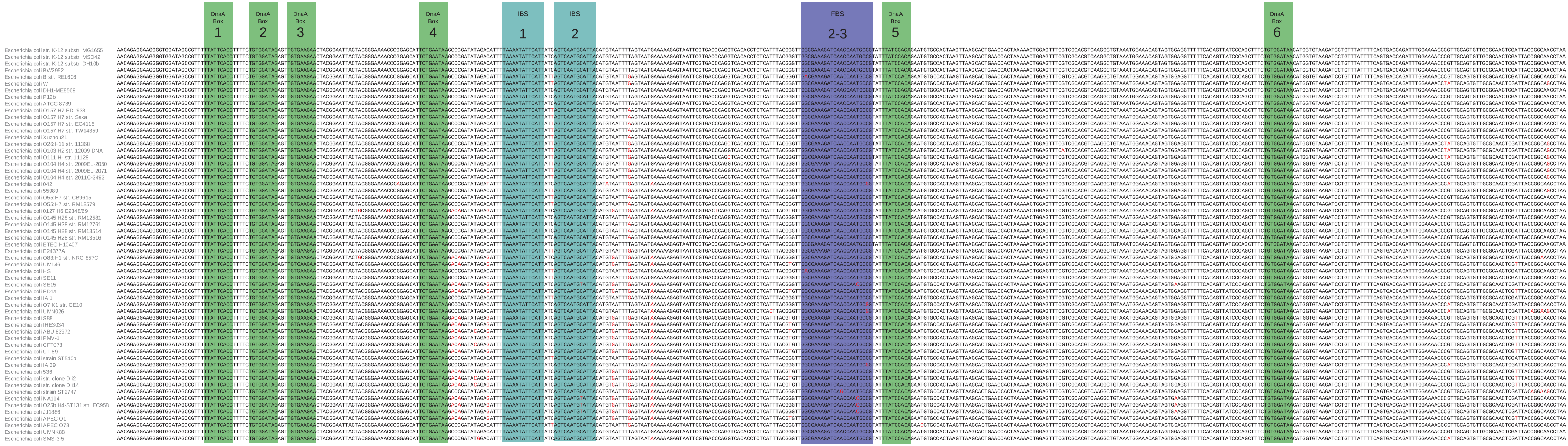

2 Supplementary Tables

Table S1. Bacterial strians

Strain	Genotype[a]	Abbreviated form	Reference/Source
MG1655	$F^{-}\lambda^{-}$ *rph-1*		(Guyer et al., 1981)
ALO4292	MG1655 strR[a]	Wild-type	This work
ALO4254	Δ*DARS2*::*cat*[a]		This work
ALO4310	Δ*DARS2*::*cat* strR[a]	Δ*DARS2*	This work
ALO4312	Δ*DARS2* strR[a]		This work
ALO4313	Δ*DARS1*::*cat* strR[a]	Δ*DARS1*	This work
ALO4315	Δ*DARS2* Δ*DARS1*::*cat* strR[a]	Δ*DARS1* Δ*DARS2*	This work
RSD428	DPB271 Δ*datA*::*kan* CAG18452		(Kitagawa et al., 1998)
ALO4331	Δ*datA*::*kan* strR[a]	Δ*datA*	This work
ALO4511	Δ*DARS2* Δ*DARS1*::*cat* Δ*datA*::*kan* strR[a]	Δ*DARS1* Δ*DARS2* Δ*datA*	This work
RB210	*E. coli* MC1000 λRB1 (*dnaA-lacZ*)		(Braun et al., 1985)
ALO1257	Δ*lacZ::Tn5*[a]		This work

ALO4618	Δ*lacZ* Δ*DARS1*[a]	This work
ALO4619	Δ*lacZ* Δ*DARS2*[a]	This work
ALO1265	Δ*lacZ::Tn5*, λRB1 (*dnaA*-*lacZ*)[a]	This work
ALO4626	Δ*lacZ::Tn5*, λRB1 (*dnaA*-*lacZ*) ΔDARS2[a]	This work
ALO4627	Δ*lacZ::Tn5*, λRB1 (*dnaA*-*lacZ*) Δ*datA*[a]	This work

[a] Genotype otherwise as MG1655.

Table S2. *E. coli* genomes with relative distances that are two times the standard deviation or more away from the *E. coli* average.

**Strain**	***DARS1***[a]	***DARS2***[a]	***dif***[a]	***datA***[a]
W3110	38	16	46	15
MC4100	47	21	30	31
ST540a	11	48	15	46
ST540b	36	20	49	11
UM146	22	31	39	10
IAI39	27	22	48	6
E. coli average	32 ± 2	21 ± 2	49 ± 2	10 ± 1

[a] Relative distance from *oriC* to indicated region; distance is in centrosomes (see Materials and Methods)

Table S3. Relative distance of *DARS1*, *DARS2*, *dif* and *datA* from *oriC*

Type	Strain	*DARS1*[a]	*DARS2*[a]	*dif*[a]	*datA*[a]	GS (bp)[b]	Ref.[c]
EHEC	O157:H7 EDL933	30	19	49	9	5528445	(Perna et al., 2001)
EHEC	O157:H7 str. Sakai	31	19	47	10	5498450	(Hayashi et al., 2001)
EHEC	O157:H7 str. EC4115	30	18	50	9	5572075	(Eppinger et al., 2011)
EHEC	O157:H7 str. TW14359	31	19	50	9	5528136	(Kulasekara et al., 2009)
EHEC	O26:H11 str. 11368	31	18	49	10	5697240	(Ogura, 2011)
EHEC	O103:H2 str. 12009 DNA	33	20	49	11	5449314	(Ogura, 2011)
EHEC	O111:H- str. 11128	31	20	50	10	5371077	(Ogura, 2011)
EHEC	Xuzhou21	31	19	48	9	5386223	(Xiong et al., 2012)
EAEC	O104:H4 str. 2009EL-2071	31	21	49	10	5312586	(Ahmed et al., 2012)
EAEC	O104:H4 str. 2011C-3493	31	22	49	10	5273097	(Ahmed et al., 2012)
EAEC	O104:H4 str. 2009EL-2050	31	22	49	10	5253138	(Ahmed et al., 2012)
EAEC	042	34	22	49	11	5241977	(Chaudhuri et al., 2010)
EAEC	55989	33	22	50	10	5154862	(Touchon et al., 2009)
EPEC	O55:H7 str. CB9615	34	20	50	9	5386352	(Zhou et al., 2010)
EPEC	O55:H7 str.	34	20	49	9	5263980	(Kyle et al., 2012)

	RM12579						
EPEC	0127:H6 E2348/69	31	20	49	10	4965553	(Iguchi et al., 2009)
STEC	O145:H28 str. RM12581	32	20	49	11	5585611	(Cooper et al., 2014)
STEC	O145:H28 str. RM12761	31	19	49	11	5402281	(De Schrijver et al., 2008)
STEC	O145:H28 str. RM13514	32	20	49	11	5585613	(Cooper et al., 2014)
STEC	O145:H28 str. RM13516	31	19	49	11	5402276	(Buvens et al., 2011)
ETEC	ETEC H10407	33	21	49	11	5153435	(Crossman et al., 2010)
ETEC	E24377A	32	22	49	10	4979619	(Rasko et al., 2008)
CD	O83:H1 str. NRG 857C	33	21	50	12	4747819	(Eaves-Pyles et al., 2008)
CD	UM146	22	31	39	10	4993013	(Krause et al., 2011)
HC	HS	33	21	49	10	4643538	(Rasko et al., 2008)
HC	SE11	33	20	49	10	4887515	(Oshima et al., 2008)
HC	SE15	34	21	50	11	4717338	(Toh et al., 2010)
HC	ED1a	31	22	48	11	5209548	(Touchon et al., 2009)
HC	IAI1	33	21	49	10	4700560	(Touchon et al., 2009)
ExPEC	O7:K1 str. CE10	32	22	49	10	5313531	(Lu et al., 2011)

ExPEC	UMN026	34	22	50	9	5202090	(Touchon et al., 2009)
ExPEC	S88	34	21	49	12	5032268	(Peigne et al., 2009)
ExPEC	IHE3034	32	19	49	10	5108383	(Moriel et al., 2010)
ExPEC	ABU 83972	33	21	50	10	5131397	(Zdziarski et al., 2010)
ExPEC	PMV-1	32	20	49	11	4984940	Direct sub.
UPEC	CFT073	31	22	49	11	5231428	(Welch et al., 2002)
UPEC	UTI89	33	20	50	9	5065741	(Chen et al., 2006)
UPEC	ST540b	36	20	49	11	4807977	(Xavier et al., 2014)
UPEC	IAI39	27	22	48	6	5132068	(Touchon et al., 2009)
UPEC	536	34	22	48	10	4938920	(Dobrindt et al., 2002)
UPEC	Clone D i2	32	22	50	10	5038386	(Reeves et al., 2011)
UPEC	Clone D i14	32	22	50	10	5038386	(Reeves et al., 2011)
UPEC	ST2747	34	20	48	12	5054509	(Xavier et al., 2014)
UPEC	NA114	35	21	48	11	4971461	(Avasthi et al., 2011)
ST131	O25b:H4-ST131 str. EC958	32	22	49	10	5109767	(Totsika et al., 2011)
ST131	JJ1886	33	21	50	10	5129938	(Andersen et al., 2013)
AP	APEC O1	32	21	49	11	5082025	(Johnson et al.,

							2006)
AP	APEC O78	35	21	49	10	4798435	(Mangiamele et al., 2013)
AP	UMNK88	31	20	50	11	5186416	(Shepard et al., 2012)
ES	SMS-3-5	33	23	50	11	5068389	(Fricke et al., 2008)
LS	MG1655	33	21	50	10	4641652	(Blattner et al., 1997)
LS	MDS42	33	22	48	12	3976195	Direct sub.
LS	DH10B	33	20	50	10	4686137	(Durfee et al., 2008)
LS	BW2952	32	21	49	11	4578159	(Ferenci et al., 2009)
LS	B str. REL606	33	22	50	10	4629812	(Jeong et al., 2009)
LS	W	33	20	50	10	4900968	(Archer et al., 2011)
LS	DH1-ME8569	33	21	50	10	4621430	(Suzuki et al., 2011)
LS	P12b	31	21	49	10	4935294	(Liu et al., 2012)
LS	ATCC 8739	33	21	50	11	4746218	Direct sub.
	Average	**32**	**21**	**49**	**10**	**5082174**	
	Standard deviation	**2**	**2**	**2**	**1**	**316847**	
	Median	**33**	**21**	**49**	**10**	**5095204**	

[a] EHEC (enterohemorrhagic *E. coli*); EAEC (enteroaggregative *E. coli*); EPEC (enteropathogenic *E. coli*); STEC (shiga toxin-producing *E. coli*); ETEC (enterotoxigenic *E. coli*); CD (Chron's disease); HC (human commensal); ExPEC (extraintestinal pathogenic *E. coli*); UPEC (uropathogenic *E. coli*); AP (animal pathogen); ES (environmental strain); LS (laboratory strain).

[b] Relative distance from *oriC* to indicated region; distance is in centrosomes (see Materials and Methods);

[c] Genome Size (GS) in base pairs;

[d] Direct submission (Direct sub.) to http://www.ebi.ac.uk/genomes/bacteria.html

References

Ahmed, S.A., Awosika, J., Baldwin, C., Bishop-Lilly, K.A., Biswas, B., Broomall, S., Chain, P.S., Chertkov, O., Chokoshvili, O., Coyne, S., Davenport, K., Detter, J.C., Dorman, W., Erkkila, T.H., Folster, J.P., Frey, K.G., George, M., Gleasner, C., Henry, M., Hill, K.K., Hubbard, K., Insalaco, J., Johnson, S., Kitzmiller, A., Krepps, M., Lo, C.C., Luu, T., Mcnew, L.A., Minogue, T., Munk, C.A., Osborne, B., Patel, M., Reitenga, K.G., Rosenzweig, C.N., Shea, A., Shen, X., Strockbine, N., Tarr, C., Teshima, H., Van Gieson, E., Verratti, K., Wolcott, M., Xie, G., Sozhamannan, S., Gibbons, H.S., and Threat Characterization, C. (2012). Genomic comparison of Escherichia coli O104:H4 isolates from 2009 and 2011 reveals plasmid, and prophage heterogeneity, including shiga toxin encoding phage stx2. *PLoS One* 7**,** e48228. doi: 10.1371/journal.pone.0048228.

Andersen, P.S., Stegger, M., Aziz, M., Contente-Cuomo, T., Gibbons, H.S., Keim, P., Sokurenko, E.V., Johnson, J.R., and Price, L.B. (2013). Complete Genome Sequence of the Epidemic and Highly Virulent CTX-M-15-Producing H30-Rx Subclone of Escherichia coli ST131. *Genome Announc* 1. doi: 10.1128/genomeA.00988-13.

Archer, C.T., Kim, J.F., Jeong, H., Park, J.H., Vickers, C.E., Lee, S.Y., and Nielsen, L.K. (2011). The genome sequence of E. coli W (ATCC 9637): comparative genome analysis and an improved genome-scale reconstruction of E. coli. *BMC Genomics* 12**,** 9. doi: 10.1186/1471-2164-12-9.

Avasthi, T.S., Kumar, N., Baddam, R., Hussain, A., Nandanwar, N., Jadhav, S., and Ahmed, N. (2011). Genome of multidrug-resistant uropathogenic Escherichia coli strain NA114 from India. *J Bacteriol* 193**,** 4272-4273. doi: 10.1128/JB.05413-11.

Blattner, F.R., Plunkett, G., 3rd, Bloch, C.A., Perna, N.T., Burland, V., Riley, M., Collado-Vides, J., Glasner, J.D., Rode, C.K., Mayhew, G.F., Gregor, J., Davis, N.W., Kirkpatrick, H.A., Goeden, M.A., Rose, D.J., Mau, B., and Shao, Y. (1997). The complete genome sequence of Escherichia coli K-12. *Science* 277**,** 1453-1462.

Braun, R.E., O'day, K., and Wright, A. (1985). Autoregulation of the DNA replication gene dnaA in E. coli K-12. *Cell* 40**,** 159-169.

Buvens, G., Posse, B., De Schrijver, K., De Zutter, L., Lauwers, S., and Pierard, D. (2011). Virulence profiling and quantification of verocytotoxin-producing Escherichia coli O145:H28 and O26:H11 isolated during an ice cream-related hemolytic uremic syndrome outbreak. *Foodborne Pathog Dis* 8**,** 421-426. doi: 10.1089/fpd.2010.0693.

Chaudhuri, R.R., Sebaihia, M., Hobman, J.L., Webber, M.A., Leyton, D.L., Goldberg, M.D., Cunningham, A.F., Scott-Tucker, A., Ferguson, P.R., Thomas, C.M., Frankel, G., Tang, C.M., Dudley, E.G., Roberts, I.S., Rasko, D.A., Pallen, M.J., Parkhill, J., Nataro, J.P., Thomson, N.R., and Henderson, I.R. (2010). Complete genome sequence and comparative metabolic profiling of the prototypical enteroaggregative Escherichia coli strain 042. *PLoS One* 5**,** e8801. doi: 10.1371/journal.pone.0008801.

Chen, S.L., Hung, C.S., Xu, J., Reigstad, C.S., Magrini, V., Sabo, A., Blasiar, D., Bieri, T., Meyer, R.R., Ozersky, P., Armstrong, J.R., Fulton, R.S., Latreille, J.P., Spieth, J., Hooton, T.M., Mardis, E.R., Hultgren, S.J., and Gordon, J.I. (2006). Identification of genes subject to positive selection in uropathogenic strains of Escherichia coli: a comparative genomics approach. *Proc Natl Acad Sci U S A* 103**,** 5977-5982. doi: 10.1073/pnas.0600938103.

Cooper, K.K., Mandrell, R.E., Louie, J.W., Korlach, J., Clark, T.A., Parker, C.T., Huynh, S., Chain, P.S., Ahmed, S., and Carter, M.Q. (2014). Complete Genome Sequences of Two Escherichia

coli O145:H28 Outbreak Strains of Food Origin. *Genome Announc* 2. doi: 10.1128/genomeA.00482-14.
Crossman, L.C., Chaudhuri, R.R., Beatson, S.A., Wells, T.J., Desvaux, M., Cunningham, A.F., Petty, N.K., Mahon, V., Brinkley, C., Hobman, J.L., Savarino, S.J., Turner, S.M., Pallen, M.J., Penn, C.W., Parkhill, J., Turner, A.K., Johnson, T.J., Thomson, N.R., Smith, S.G., and Henderson, I.R. (2010). A commensal gone bad: complete genome sequence of the prototypical enterotoxigenic Escherichia coli strain H10407. *J Bacteriol* 192**,** 5822-5831. doi: 10.1128/JB.00710-10.
De Schrijver, K., Buvens, G., Posse, B., Van Den Branden, D., Oosterlynck, O., De Zutter, L., Eilers, K., Pierard, D., Dierick, K., Van Damme-Lombaerts, R., Lauwers, C., and Jacobs, R. (2008). Outbreak of verocytotoxin-producing E. coli O145 and O26 infections associated with the consumption of ice cream produced at a farm, Belgium, 2007. *Euro Surveill* 13.
Dobrindt, U., Emody, L., Gentschev, I., Goebel, W., and Hacker, J. (2002). Efficient expression of the alpha-haemolysin determinant in the uropathogenic Escherichia coli strain 536 requires the leuX-encoded tRNA(5)(Leu). *Mol Genet Genomics* 267**,** 370-379. doi: 10.1007/s00438-002-0668-3.
Durfee, T., Nelson, R., Baldwin, S., Plunkett, G., 3rd, Burland, V., Mau, B., Petrosino, J.F., Qin, X., Muzny, D.M., Ayele, M., Gibbs, R.A., Csorgo, B., Posfai, G., Weinstock, G.M., and Blattner, F.R. (2008). The complete genome sequence of Escherichia coli DH10B: insights into the biology of a laboratory workhorse. *J Bacteriol* 190**,** 2597-2606. doi: 10.1128/JB.01695-07.
Eaves-Pyles, T., Allen, C.A., Taormina, J., Swidsinski, A., Tutt, C.B., Jezek, G.E., Islas-Islas, M., and Torres, A.G. (2008). Escherichia coli isolated from a Crohn's disease patient adheres, invades, and induces inflammatory responses in polarized intestinal epithelial cells. *Int J Med Microbiol* 298**,** 397-409. doi: 10.1016/j.ijmm.2007.05.011.
Eppinger, M., Mammel, M.K., Leclerc, J.E., Ravel, J., and Cebula, T.A. (2011). Genomic anatomy of Escherichia coli O157:H7 outbreaks. *Proc Natl Acad Sci U S A* 108**,** 20142-20147. doi: 10.1073/pnas.1107176108.
Ferenci, T., Zhou, Z., Betteridge, T., Ren, Y., Liu, Y., Feng, L., Reeves, P.R., and Wang, L. (2009). Genomic sequencing reveals regulatory mutations and recombinational events in the widely used MC4100 lineage of Escherichia coli K-12. *J Bacteriol* 191**,** 4025-4029. doi: 10.1128/JB.00118-09.
Fricke, W.F., Wright, M.S., Lindell, A.H., Harkins, D.M., Baker-Austin, C., Ravel, J., and Stepanauskas, R. (2008). Insights into the environmental resistance gene pool from the genome sequence of the multidrug-resistant environmental isolate Escherichia coli SMS-3-5. *J Bacteriol* 190**,** 6779-6794. doi: 10.1128/JB.00661-08.
Guyer, M.S., Reed, R.R., Steitz, J.A., and Low, K.B. (1981). Identification of a sex-factor-affinity site in E. coli as gamma delta. *Cold Spring Harb Symp Quant Biol* 45 Pt 1**,** 135-140.
Hayashi, T., Makino, K., Ohnishi, M., Kurokawa, K., Ishii, K., Yokoyama, K., Han, C.G., Ohtsubo, E., Nakayama, K., Murata, T., Tanaka, M., Tobe, T., Iida, T., Takami, H., Honda, T., Sasakawa, C., Ogasawara, N., Yasunaga, T., Kuhara, S., Shiba, T., Hattori, M., and Shinagawa, H. (2001). Complete genome sequence of enterohemorrhagic Escherichia coli O157:H7 and genomic comparison with a laboratory strain K-12. *DNA Res* 8**,** 11-22.
Iguchi, A., Thomson, N.R., Ogura, Y., Saunders, D., Ooka, T., Henderson, I.R., Harris, D., Asadulghani, M., Kurokawa, K., Dean, P., Kenny, B., Quail, M.A., Thurston, S., Dougan, G., Hayashi, T., Parkhill, J., and Frankel, G. (2009). Complete genome sequence and comparative genome analysis of enteropathogenic Escherichia coli O127:H6 strain E2348/69. *J Bacteriol* 191**,** 347-354. doi: 10.1128/JB.01238-08.

Jeong, H., Barbe, V., Lee, C.H., Vallenet, D., Yu, D.S., Choi, S.H., Couloux, A., Lee, S.W., Yoon, S.H., Cattolico, L., Hur, C.G., Park, H.S., Segurens, B., Kim, S.C., Oh, T.K., Lenski, R.E., Studier, F.W., Daegelen, P., and Kim, J.F. (2009). Genome sequences of Escherichia coli B strains REL606 and BL21(DE3). *J Mol Biol* 394**,** 644-652. doi: 10.1016/j.jmb.2009.09.052.

Johnson, T.J., Johnson, S.J., and Nolan, L.K. (2006). Complete DNA sequence of a ColBM plasmid from avian pathogenic Escherichia coli suggests that it evolved from closely related ColV virulence plasmids. *J Bacteriol* 188**,** 5975-5983. doi: 10.1128/JB.00204-06.

Kitagawa, R., Ozaki, T., Moriya, S., and Ogawa, T. (1998). Negative control of replication initiation by a novel chromosomal locus exhibiting exceptional affinity for Escherichia coli DnaA protein. *Genes Dev* 12**,** 3032-3043.

Krause, D.O., Little, A.C., Dowd, S.E., and Bernstein, C.N. (2011). Complete genome sequence of adherent invasive Escherichia coli UM146 isolated from Ileal Crohn's disease biopsy tissue. *J Bacteriol* 193**,** 583. doi: 10.1128/JB.01290-10.

Kulasekara, B.R., Jacobs, M., Zhou, Y., Wu, Z., Sims, E., Saenphimmachak, C., Rohmer, L., Ritchie, J.M., Radey, M., Mckevitt, M., Freeman, T.L., Hayden, H., Haugen, E., Gillett, W., Fong, C., Chang, J., Beskhlebnaya, V., Waldor, M.K., Samadpour, M., Whittam, T.S., Kaul, R., Brittnacher, M., and Miller, S.I. (2009). Analysis of the genome of the Escherichia coli O157:H7 2006 spinach-associated outbreak isolate indicates candidate genes that may enhance virulence. *Infect Immun* 77**,** 3713-3721. doi: 10.1128/IAI.00198-09.

Kyle, J.L., Cummings, C.A., Parker, C.T., Quinones, B., Vatta, P., Newton, E., Huynh, S., Swimley, M., Degoricija, L., Barker, M., Fontanoz, S., Nguyen, K., Patel, R., Fang, R., Tebbs, R., Petrauskene, O., Furtado, M., and Mandrell, R.E. (2012). Escherichia coli serotype O55:H7 diversity supports parallel acquisition of bacteriophage at Shiga toxin phage insertion sites during evolution of the O157:H7 lineage. *J Bacteriol* 194**,** 1885-1896. doi: 10.1128/JB.00120-12.

Liu, B., Hu, B., Zhou, Z., Guo, D., Guo, X., Ding, P., Feng, L., and Wang, L. (2012). A novel non-homologous recombination-mediated mechanism for Escherichia coli unilateral flagellar phase variation. *Nucleic Acids Res* 40**,** 4530-4538. doi: 10.1093/nar/gks040.

Lu, S., Zhang, X., Zhu, Y., Kim, K.S., Yang, J., and Jin, Q. (2011). Complete genome sequence of the neonatal-meningitis-associated Escherichia coli strain CE10. *J Bacteriol* 193**,** 7005. doi: 10.1128/JB.06284-11.

Mangiamele, P., Nicholson, B., Wannemuehler, Y., Seemann, T., Logue, C.M., Li, G., Tivendale, K.A., and Nolan, L.K. (2013). Complete genome sequence of the avian pathogenic Escherichia coli strain APEC O78. *Genome Announc* 1**,** e0002613. doi: 10.1128/genomeA.00026-13.

Moriel, D.G., Bertoldi, I., Spagnuolo, A., Marchi, S., Rosini, R., Nesta, B., Pastorello, I., Corea, V.A., Torricelli, G., Cartocci, E., Savino, S., Scarselli, M., Dobrindt, U., Hacker, J., Tettelin, H., Tallon, L.J., Sullivan, S., Wieler, L.H., Ewers, C., Pickard, D., Dougan, G., Fontana, M.R., Rappuoli, R., Pizza, M., and Serino, L. (2010). Identification of protective and broadly conserved vaccine antigens from the genome of extraintestinal pathogenic Escherichia coli. *Proc Natl Acad Sci U S A* 107**,** 9072-9077. doi: 10.1073/pnas.0915077107.

Ogura, Y. (2011). [Genomic analyses of mechanisms of virulence evolution in enterohemorrhagic E. coli and enteropathogenic E. coli]. *Nihon Saikingaku Zasshi* 66**,** 175-186.

Oshima, K., Toh, H., Ogura, Y., Sasamoto, H., Morita, H., Park, S.H., Ooka, T., Iyoda, S., Taylor, T.D., Hayashi, T., Itoh, K., and Hattori, M. (2008). Complete genome sequence and comparative analysis of the wild-type commensal Escherichia coli strain SE11 isolated from a healthy adult. *DNA Res* 15**,** 375-386. doi: 10.1093/dnares/dsn026.

Peigne, C., Bidet, P., Mahjoub-Messai, F., Plainvert, C., Barbe, V., Medigue, C., Frapy, E., Nassif, X., Denamur, E., Bingen, E., and Bonacorsi, S. (2009). The plasmid of Escherichia coli strain S88 (O45:K1:H7) that causes neonatal meningitis is closely related to avian pathogenic E. coli plasmids and is associated with high-level bacteremia in a neonatal rat meningitis model. *Infect Immun* 77**,** 2272-2284. doi: 10.1128/IAI.01333-08.

Perna, N.T., Plunkett, G., 3rd, Burland, V., Mau, B., Glasner, J.D., Rose, D.J., Mayhew, G.F., Evans, P.S., Gregor, J., Kirkpatrick, H.A., Posfai, G., Hackett, J., Klink, S., Boutin, A., Shao, Y., Miller, L., Grotbeck, E.J., Davis, N.W., Lim, A., Dimalanta, E.T., Potamousis, K.D., Apodaca, J., Anantharaman, T.S., Lin, J., Yen, G., Schwartz, D.C., Welch, R.A., and Blattner, F.R. (2001). Genome sequence of enterohaemorrhagic Escherichia coli O157:H7. *Nature* 409**,** 529-533. doi: 10.1038/35054089.

Rasko, D.A., Rosovitz, M.J., Myers, G.S., Mongodin, E.F., Fricke, W.F., Gajer, P., Crabtree, J., Sebaihia, M., Thomson, N.R., Chaudhuri, R., Henderson, I.R., Sperandio, V., and Ravel, J. (2008). The pangenome structure of Escherichia coli: comparative genomic analysis of E. coli commensal and pathogenic isolates. *J Bacteriol* 190**,** 6881-6893. doi: 10.1128/JB.00619-08.

Reeves, P.R., Liu, B., Zhou, Z., Li, D., Guo, D., Ren, Y., Clabots, C., Lan, R., Johnson, J.R., and Wang, L. (2011). Rates of mutation and host transmission for an Escherichia coli clone over 3 years. *PLoS One* 6**,** e26907. doi: 10.1371/journal.pone.0026907.

Shepard, S.M., Danzeisen, J.L., Isaacson, R.E., Seemann, T., Achtman, M., and Johnson, T.J. (2012). Genome sequences and phylogenetic analysis of K88- and F18-positive porcine enterotoxigenic Escherichia coli. *J Bacteriol* 194**,** 395-405. doi: 10.1128/JB.06225-11.

Suzuki, S., Ono, N., Furusawa, C., Ying, B.W., and Yomo, T. (2011). Comparison of sequence reads obtained from three next-generation sequencing platforms. *PLoS One* 6**,** e19534. doi: 10.1371/journal.pone.0019534.

Toh, H., Oshima, K., Toyoda, A., Ogura, Y., Ooka, T., Sasamoto, H., Park, S.H., Iyoda, S., Kurokawa, K., Morita, H., Itoh, K., Taylor, T.D., Hayashi, T., and Hattori, M. (2010). Complete genome sequence of the wild-type commensal Escherichia coli strain SE15, belonging to phylogenetic group B2. *J Bacteriol* 192**,** 1165-1166. doi: 10.1128/JB.01543-09.

Totsika, M., Beatson, S.A., Sarkar, S., Phan, M.D., Petty, N.K., Bachmann, N., Szubert, M., Sidjabat, H.E., Paterson, D.L., Upton, M., and Schembri, M.A. (2011). Insights into a multidrug resistant Escherichia coli pathogen of the globally disseminated ST131 lineage: genome analysis and virulence mechanisms. *PLoS One* 6**,** e26578. doi: 10.1371/journal.pone.0026578.

Touchon, M., Hoede, C., Tenaillon, O., Barbe, V., Baeriswyl, S., Bidet, P., Bingen, E., Bonacorsi, S., Bouchier, C., Bouvet, O., Calteau, A., Chiapello, H., Clermont, O., Cruveiller, S., Danchin, A., Diard, M., Dossat, C., Karoui, M.E., Frapy, E., Garry, L., Ghigo, J.M., Gilles, A.M., Johnson, J., Le Bouguenec, C., Lescat, M., Mangenot, S., Martinez-Jehanne, V., Matic, I., Nassif, X., Oztas, S., Petit, M.A., Pichon, C., Rouy, Z., Ruf, C.S., Schneider, D., Tourret, J., Vacherie, B., Vallenet, D., Medigue, C., Rocha, E.P., and Denamur, E. (2009). Organised genome dynamics in the Escherichia coli species results in highly diverse adaptive paths. *PLoS Genet* 5**,** e1000344. doi: 10.1371/journal.pgen.1000344.

Welch, R.A., Burland, V., Plunkett, G., 3rd, Redford, P., Roesch, P., Rasko, D., Buckles, E.L., Liou, S.R., Boutin, A., Hackett, J., Stroud, D., Mayhew, G.F., Rose, D.J., Zhou, S., Schwartz, D.C., Perna, N.T., Mobley, H.L., Donnenberg, M.S., and Blattner, F.R. (2002). Extensive mosaic structure revealed by the complete genome sequence of uropathogenic Escherichia coli. *Proc Natl Acad Sci U S A* 99**,** 17020-17024. doi: 10.1073/pnas.252529799.

Xavier, B.B., Vervoort, J., Stewardson, A., Adriaenssens, N., Coenen, S., Harbarth, S., Goossens, H., and Malhotra-Kumar, S. (2014). Complete Genome Sequences of Nitrofurantoin-Sensitive

and -Resistant Escherichia coli ST540 and ST2747 Strains. *Genome Announc* 2. doi: 10.1128/genomeA.00239-14.
Xiong, Y., Wang, P., Lan, R., Ye, C., Wang, H., Ren, J., Jing, H., Wang, Y., Zhou, Z., Bai, X., Cui, Z., Luo, X., Zhao, A., Wang, Y., Zhang, S., Sun, H., Wang, L., and Xu, J. (2012). A novel Escherichia coli O157:H7 clone causing a major hemolytic uremic syndrome outbreak in China. *PLoS One* 7**,** e36144. doi: 10.1371/journal.pone.0036144.
Zdziarski, J., Brzuszkiewicz, E., Wullt, B., Liesegang, H., Biran, D., Voigt, B., Gronberg-Hernandez, J., Ragnarsdottir, B., Hecker, M., Ron, E.Z., Daniel, R., Gottschalk, G., Hacker, J., Svanborg, C., and Dobrindt, U. (2010). Host imprints on bacterial genomes--rapid, divergent evolution in individual patients. *PLoS Pathog* 6**,** e1001078. doi: 10.1371/journal.ppat.1001078.
Zhou, Z., Li, X., Liu, B., Beutin, L., Xu, J., Ren, Y., Feng, L., Lan, R., Reeves, P.R., and Wang, L. (2010). Derivation of Escherichia coli O157:H7 from its O55:H7 precursor. *PLoS One* 5**,** e8700. doi: 10.1371/journal.pone.0008700.
